# Multiomics Reveal Associations Between CpG Methylation, Histone Modifications and Transcription in a Species That has Lost DNMT3, the Colorado Potato Beetle

**DOI:** 10.1002/jez.b.23303

**Published:** 2025-05-12

**Authors:** Zoe M. Länger, Elisa Israel, Jan Engelhardt, Agata I. Kalita, Claudia I. Keller Valsecchi, Joachim Kurtz, Sonja J. Prohaska

**Affiliations:** ^1^ Institute for Evolution and Biodiversity (IEB) University of Münster Münster Germany; ^2^ Computational EvoDevo Group, Institute of Computer Science Leipzig University Leipzig Germany; ^3^ Department of Evolutionary Biology University of Vienna Vienna Austria; ^4^ Institute of Molecular Biology (IMB) Mainz Germany; ^5^ Joint Institute for Individualisation in a Changing Environment (JICE) University of Münster and Bielefeld University Münster Germany

**Keywords:** Coleoptera, CUT&Tag, EM‐seq, H3K27ac, H3K36me3, RNA‐seq

## Abstract

Insects display exceptional phenotypic plasticity, which can be mediated by epigenetic modifications, including CpG methylation and histone modifications. In vertebrates, both are interlinked and CpG methylation is associated with gene repression. However, little is known about these regulatory systems in invertebrates, where CpG methylation is mainly restricted to gene bodies of transcriptionally active genes. A widely conserved mechanism involves the co‐transcriptional deposition of H3K36 trimethylation and the targeted methylation of unmethylated CpGs by the *de novo* DNA methyltransferase DNMT3. However, DNMT3 has been lost multiple times in invertebrate lineages raising the question of how the links between CpG methylation, histone modifications and gene expression are affected by its loss. Here, we report the epigenetic landscape of *Leptinotarsa decemlineata*, a beetle species that has lost DNMT3 but retained CpG methylation. We combine RNA‐seq, enzymatic methyl‐seq and CUT&Tag to study gene expression, CpG methylation and patterns of H3K36me3 and H3K27ac histone modifications on a genome‐wide scale. Despite the loss of DNMT3, H3K36me3 mirrors CpG methylation patterns. Together, they give rise to signature profiles for expressed and not expressed genes. H3K27ac patterns show a prominent peak at the transcription start site that is predictive of expressed genes irrespective of their methylation status. Our study provides new insights into the evolutionary flexibility of epigenetic modification systems that urge caution when generalizing across species.

## Graphical abstract

Despite lacking DNMT3, EM‐seq revealed CpG methylation in the Colorado potato beetle. CUT&Tag analysis showed an association of H3K36me3 and H3K27ac with transcription, with H3K36me3 mirroring CpG methylation, demonstrating epigenetic flexibility.

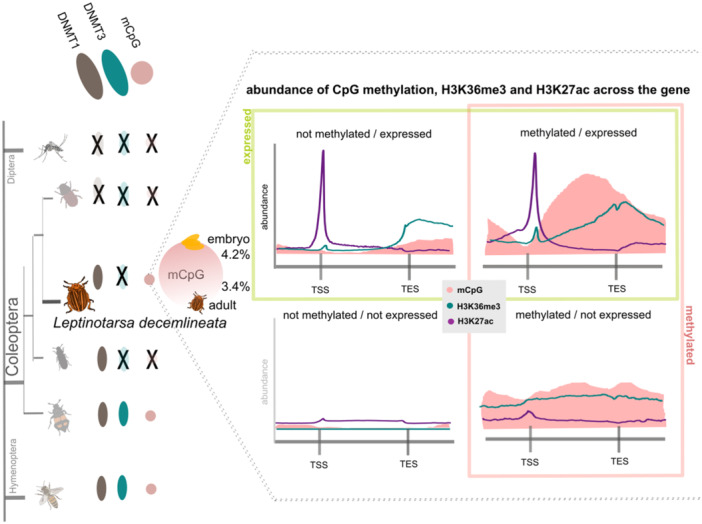

## 1 Introduction

The remarkable evolutionary success of insects is largely due to their exceptional ability to generate phenotypic diversity. The basis for such plasticity is partially provided by epigenetic modifications, which facilitate differential gene expression. This is achieved through reversible chemical modifications on histones or DNA bases (Aliaga et al. 2019). While epigenetic modifications as such are well conserved, their functions and interconnections might be evolutionarily flexible. However, we currently know little about this beyond a few intensively studied model organisms.

The most prominent DNA methylation involves the addition of a methyl group to position 5 of cytosine in a CpG dinucleotide context (Lister et al. 2009). This modification is established *de novo* by DNA methyltransferase 3 (DNMT3) and faithfully maintained by DNMT1 during DNA replication (Lyko 2017). In vertebrates, CpG methylation is present at medium to high levels and is mainly associated with gene silencing (Bourc'his and Bestor 2004; Greenberg and Bourc'his 2019). Conversely, in invertebrates, CpG methylation predominantly occurs in gene bodies, especially in exons of active genes as reported for multiple species, including the honey bee (*Apis mellifera*) and the silk moth (*Bombyx mori*) (Lewis et al. 2020; Suzuki et al. 2007; Zemach et al. 2010). However, changes in methylation do not seem to lead to coordinated changes in transcription (Cardoso‐Júnior et al. 2021; Dixon and Matz 2022; Harris et al. 2019), although this is still debated in social insects, especially with regard to caste differentiation (Kucharski et al. 2008; Oldroyd and Yagound 2021; Marshall et al. 2023; Okwaro and Korb 2023).

Many invertebrate model organisms, such as *Drosophila melanogaster* and *Caenorhabditis elegans*, lack detectable levels of CpG methylation (Raddatz et al. 2013; Zemach et al. 2010), reflecting the recurrent loss of DNA methylation throughout Ecdysozoan evolution (Engelhardt et al. 2022), and casting doubt on the central role of CpG methylation in gene regulation. In Coleoptera, some species have lost CpG methylation (e.g., *Tribolium castaneum* (Schulz et al. 2018)), while others, like *Nicrophorus vespilloides*, retained low levels (Cunningham et al. 2015). The focal association of gene body methylation with actively transcribed genes and the overall reduction of CpG methylation in holometabolous insects suggest divergent functional evolution of CpG methylation in vertebrates and invertebrates (Hunt et al. 2013; Provataris et al. 2018). Many species with low CpG methylation have lost DNMT3 but retained DNMT1 (Engelhardt et al. 2022). This may suggest that DNMT1 alone is maintaining CpG methylation over many generations, or acts as a *de novo* methyltransferase under certain conditions (Glastad et al. 2019; Yarychkivska et al. 2018).

Modifications on histones can alter the structure of chromatin and affect various parts of the gene transcription process, which can lead to correlations between histone modification and gene expression (Zhang et al. 2022). A core set of histone modifications is broadly conserved across eukaryotes, including H3 and H4 lysine acetylation marks, indicative of gene expression‐permissive chromatin and H3 lysine trimethylation marks, which demarcate either active (e.g., H3K4me3 and H3K36me3) or repressive (e.g., H3K27me3 and H3K9me3) chromatin states (Grau‐Bové et al. 2022). In particular, H3K36me3 is prevalent on gene bodies of active genes and associated with transcription (Guenther et al. 2007; Pokholok et al. 2005). In contrast, acetylation of histone H3 lysine 27 (H3K27ac) is commonly associated with regulatory regions, that is, promoters and enhancers, in the context of gene activity (Nègre et al. 2011; Simola et al. 2013). H3K27ac is involved in nucleosome mobilization and eviction (Kang et al. 2021), at the transcription start site (TSS) of active genes. Furthermore, H3K27ac, set by homologs of the histone acetyltransferase p300/CBP, antagonizes the establishment of H3K27me3, the repressive mark set by polycomb complex 2 (PRC2) (Tie et al. 2009).

In vertebrates, DNA methylation and histone modification are highly interlinked. An association of CpG methylation and H3K36 methylation appears to be deeply conserved among eukaryotes (de Mendoza et al. 2020; Hahn et al. 2011; Neri et al. 2017; Pokholok et al. 2005; Yano et al. 2022), but studies in insects showing this association are limited. Gene body methylation in *A. mellifera* and *Solenopsis invicta* (fire ant) positively correlates with activating histone marks in *D. melanogaster*, for example, H3K36me3 (Hunt et al. 2013; Nanty et al. 2011), even though *D. melanogaster* itself has lost CpG methylation. A link between H3K27ac and CpG methylation, mediated by the methyl‐CpG‐binding domain protein 2/3 (MBD2/3), was described by Xu et al. (2021) in *B. mori*. The authors propose that MBD2/3 targets Tip60, a histone H3K27 acetyltransferase complex, to methylated CpGs.

DNMTs contain domains for recognition of histone modification marks and, vice versa, histone modifying complexes and transcription factors distinguish between methylated and unmethylated CpGs. While maintenance of CpG methylation by DNMT1 is coupled to DNA replication, *de novo* establishment by DNMT3 is promoted by transcription. Here, SETD2 interacts with the elongating RNA‐Pol II to deposit H3K36me3 at the gene body (Edmunds et al. 2007; Yoh et al. 2008). In turn, DNMT3 binds to H3K36me3 via its PWWP domain and methylates unmethylated CpGs (Dhayalan et al. 2010; Teissandier and Bourc'his 2017; Wagner and Carpenter 2012). The functional overlap between CpG methylation and other epigenetic mechanisms, such as certain histone modifications, may range from perfect complementarity – rendering methylation indispensable – to full redundancy, which would facilitate the loss of CpG methylation (Hunt et al. 2013).

For species that lost DNMT3, the question arises of how CpG methylation marks can be set *de novo* to genes that become active later in development. Experimental data on DNA methylation in Coleoptera is currently only available for two species, *T. castaneum* (loss of DNMT3 and DNA methylation) and *N. vespilloides* (DNMT1, DNMT3 and DNA methylation present) (Cunningham et al. 2015; Schulz et al. 2018). We thus studied the Colorado potato beetle (*L. decemlineata)* as the first beetle species, where DNA methylation was predicted to be retained despite the loss of DNMT3 (Engelhardt et al. 2022).

This study aims to expand our knowledge of the organization of epigenetic systems in insects, providing a basis for further research leading to an in‐depth understanding of the underlying mechanisms, that is needed to appraise the epigenetic contribution to phenotypic diversity and evolutionary novelty (Maleszka 2024). Here, we experimentally assess and compare CpG methylation of *L. decemlineata* in adults and embryos, the genomic location of H3K36me3 and H3K27ac in embryos, and link that epigenetic information to genome‐wide gene expression data. We combined RNA sequencing with novel techniques for studying CpG methylation and histone modifications, enzymatic methyl‐seq (EM‐seq) and CUT&Tag, respectively, demonstrating the potential of whole‐genome approaches even in species whose epigenomes were previously largely unstudied.

## 2 Materials and Methods

### 2.1 Model Organism and Samples

We used Colorado potato beetles, *L. decemlineata* (Coleoptera). For detailed rearing conditions see supplementary methods. For replicates of embryo EM‐seq, RNA‐seq and CUT&Tag, we pooled approximately 30 embryos from different egg clusters laid on the same day. For adult sample preparation, we pooled two individuals per replicate. We sampled the living animals of approximately the same age (2–3 weeks after eclosion) on the same day for sequencing and stored them at −80°C until processing, if necessary. For EM‐seq and RNA‐seq, we used three replicates for each life stage (mixed sex). For CUT&Tag, we used two embryo replicates.

We used the chromosome‐level genome assembly and gene annotation of *L. decemlineata* provided by the Gene Expression Atlas of the Colorado Potato Beetle, denoted as version LdNA_01 (Wilhelm et al. 2025). The assembly is identical to ASM2471293v1/GCA_024712935.1 (Yan et al. 2023) on NCBI/GenBank, respectively.

### 2.2 DNA Methylation: Enzymatic Methyl Sequencing (EM‐Seq)

High molecular weight DNA was extracted from pooled whole body embryo or adult *L. decemlineata* using a combination of chloroform:isoamyl and a salting out procedure. Detailed protocol information can be found in the supplementary methods. Enzymatic methyl library preparation and sequencing were kindly performed in the Cologne Center for Genomics (CCG) University of Cologne, Cologne, Germany. The enzymatic conversion method (EM‐seq) was used, as described in (Vaisvila et al. 2021). Methylated (CpG methylated pUC19) and unmethylated (unmethylated Lambda) controls were included in the library preparation and we checked the conversion rate for these controls after sequencing. Briefly, 200 ng high molecular weight DNA was processed using NEBNext UltraShear, following the manufacturer's instructions. For a fragment length of 250–350 bp, Enzymatic fragmentation was conducted for 20 min at 37°C and 15 min at 65°C to hold at 4°C. For PCR library amplification, the following program was used: (1. 30"‐ 98°C, 2. 10"‐ 98°C, 3. 30"‐ 62°C, 4. 1'‐ 65°C (2.‐ 4. × 4), 5. 5‐ 65°C). Amplified libraries were cleaned with 65 µL of resuspended NEBNext Sample Purification Beads. Libraries were sequenced on an Illumina NextSeq. 2000 (2 × 150 bases).

### 2.3 Data Processing (EM‐Seq)

We trimmed the reads using trim_galore (Krueger et al. 2023; Martin 2011) in paired‐end mode. We used default settings but trimmed 10 nucleotides from both sides of each read (–clip_R1 = 10, –clip_R2 = 10, –three_prime_clip_R1 = 10, –three_prime_clip_R2 = 10) to remove potential remnants of EM‐seq adapters. The sequence of the DNA controls Lambda and pUC19 were downloaded from New England Biolabs and added as additional fasta entries to the assembly file. Subsequently, the reads were mapped, deduplicated, and the methylation level extracted using the Bismark pipeline (Krueger and Andrews 2011). The number of reads and coverage can be found in Table S1. To accommodate the lower quality of the *L. decemlineata* genome compared to vertebrate model organisms and the potentially larger divergence of our population from the sequenced strain, we used a lower minimum alignment score (‐‐score_min L,0,−0.6). The conversion efficiency was calculated by subtracting the relative amount of methylated cytosines in the unmethylated control Lambda from 100. The conversion efficiency ranges from 99.23% to 99.68%, see Table S2. In subsequent analyses, reads mapping to the controls were not considered. The enrichment of methylation levels in different parts of the genome was calculated using BEDTools (Krueger and Andrews 2011; Quinlan and Hall 2010) and custom shell scripts. To estimate CpG methylation levels, we require cytosines (in a CpG context) to be covered by five or more reads. We then define the methylation level of a cytosine as the fraction of the number of methylated reads divided by the total number of reads covering that cytosine. To estimate the degree of methylation of a genomic feature, such as a gene, we calculate the mean percent methylation of all CpGs within that feature, if and only if the feature contains three or more CpGs. The consolidated set of genes is generated by excluding all genes that have less than three CpGs per gene in one or more of the six samples (3x embryo and 3x adult). All replicates correlate strongly (Pearson, *p* < 0.05) regarding all individual CpG sites as well as in the methylation levels of genes (see Figures S1–4). We used RepeatModeller v2.0.5 (Flynn et al. 2020) to generate models of de novo repeats and RepeatMasker v4.1.7‐p1 to annotate them in the genome.

We used metilene version 0.2–8 (Jühling et al. 2016) to predict differentially methylated regions (DMRs) between the adult and embryo replicates. Only CpGs with five or more and less than or equal to 100 reads in all six replicates were used as input. A DMR was considered significant if it had a Benjamini‐Hochberg adjusted *p*‐value < 0.05. The resulting DMRs have at least 10 CpGs and a mean methylation difference of at least 10%.

### 2.4 Gene Expression: RNA Extraction and RNA‐Seq

Pooled whole‐body samples of embryos or adults were taken for RNA extraction. We used a protocol combining Trizol lysis and chloroform extraction with the purification via spin columns from the SV Total RNA Isolation System (Promega). The detailed protocol can be found in the supplementary methods.

### 2.5 Data Processing (RNA‐Seq)

RNA sequencing and basic data processing were carried out by Novogene (Planneg, Munich, Germany). In short samples were sequenced using the Illumina NovaSeq PE150 platform. The raw data were cleaned to remove reads containing adapters, more than 10% N's or when low quality nucleotides constituted more than 50% of the read. The number of reads and coverage can be found in Table S3. Subsequently, HISAT2 version 2.0.5 (Mortazavi et al. 2008) was used to align the reads to the genome and DESeq. 2 version 1.20.0 (Love et al. 2014) with Benjamin Hochberg adjusted *p*‐value to predict differentially expressed genes.

One embryonic replicate (embryo 1) did neither correlate (Pearson correlation) nor cluster (Principal component analysis) with the other embryonic or adult replicates (see Figures S5–7). Thus, we dismissed it in further analyses and rerun DESeq. 2, using NovoMagic, a free Novogene platform for data analysis.

### 2.6 Histone Modifications: CUT&Tag Library Generation and Sequencing

We performed CUT&Tag as described in (Kaya‐Okur et al. 2020) with slight modifications. Whole‐body samples of the studied species were flash‐frozen, homogenized in cold DPBS (supplemented with MgCl_2_ and CaCl_2_), and passed through a cell strainer (Corning, 352235). We estimated cell numbers using a Bürker chamber (cells were stained with 1x DAPI (1:1000); supplemented with 0.1% digitonin) to later equalize the cell numbers per sample by adequate dilution.

We used 0.4 million cells per reaction from whole‐body samples of the studied species. Cells were bound to ConA beads in a 1:10 ratio for 10 min at room temperature. We then incubated the cells in an antibody buffer with the primary antibody (1:100) [IgG control Rabbit (Abcam catalog no. ab37415), H3K36me3 (Active Motif catalog no. 91266), H3K27ac (Abcam catalog no. ab4729)] at 4°C overnight on a nutator, which was followed by incubation with the secondary antibodies (1:100) [αMs IgG Rabbit (Abcam catalog no. ab6709), αRb IgG Guinea pig (Sigma‐Aldrich catalog no. SAB3700890)] for 60 min at room temperature. Cells were rinsed, washed twice, and incubated with loaded pA‐Tn5 (1:200) for 1 h at room temperature on a nutator. To remove excess pA‐Tn5, cells were rinsed and then washed. To perform the tagmentation, we incubated the cells with 10 mM MgCl_2_ for 1 h at 37°C. To stop the tagmentation and solubilize the DNA fragments, we added EDTA, SDS and Proteinase K and incubated for 1 h at 55°C. Libraries were amplified with the NEBNext Ultra Q5 Master Mix (NEB) in 14 cycles and purified using the DNA Clean & Concentrator‐5 Zymo kit, following the manufacturer's instructions. Amplified libraries were resuspended in 15 µL nuclease free H_2_O. We quantified library concentrations using Qubit and measured the library size on Bioanalyzer.

Pooled samples were sequenced on Illumina NextSeq 500 High Output, PE for 2 × 75 cycles plus 2 × 8 cycles for the dual index read by the Institute for Molecular Biology, Mainz, Genomics Core Facility. pA–Tn5 was prepared by the Institute for Molecular Biology, Mainz, Protein Production Core Facility.

### 2.7 Data Processing (CUT&Tag)

We removed adapters using cutadapt version 4.8 (Martin 2011). We followed the protocol of Zheng et al. (2020) up to peak calling. With bowtie2 (version 2.5.3) (Langmead and Salzberg 2012), we aligned the trimmed reads to the reference genome of *L. decemlineata*, with the following parameters ‐‐end‐to‐end ‐‐very‐sensitive ‐‐no‐mixed ‐‐no‐discordant ‐‐phred33 ‐I 10 ‐X 700. The number of reads and alignment rate can be found in the Table S4, fragment length in Figure S8. We calculated a Pearson correlation between the replicates and samples (see Figure S9) with a custom R script. To assess the coverage of features, we employed bedtools (version 2.31.1) genomecov (Quinlan and Hall 2010). For peak calling and sparse enrichment analysis, we used SEACR version 1.3 (Meers et al. 2019) with parameters set to ‘norm’ and ‘stringent’. SEACR was given the parameter ‘0.025’ to obtain the 2.5% of highest peaks for each replicate. Subsequently, we examined the sets for overlaps using bedtools intersect and merged the resulting subset of overlapping peaks with bedtools merge. This way, we obtained a high‐confidence set of reproducible peaks. We visualized heatmaps using deepTools version 3.5.5 (Ramírez et al. 2016), employing the functions computeMatrix ‐‐scale‐regions and plotHeatmap. For this, we normalized the gene length to a length of 5 kb, with 3 kb upstream and downstream of the gene body.

### 2.8 Gene Ontology (GO) Enrichment Analysis

Gene Ontology (GO) enrichment analysis was conducted using the topGO package version 2.56.0 (Alexa and Rahnenfuhrer 2024) in R. We made use of GO term assignments from the Colorado potato beetle gene expression atlas provided by Wilhelm et al. (2025). We used the Parent‐Child algorithm to determine the highest common GO term level and identified significantly enriched GO terms using the classic Fisher's exact and corrected the *p*‐value for multiple tests using the Benjamini‐Hochberg method. Gene ratio was calculated as the percentage of genes annotated with the respective GO term in a subset to the entity of genes with the respective GO term in the genome. The 40 most significant GO terms were visualized using ggplot2 (Wickham et al. 2007).

### 2.9 Data Visualization

Part of the data was analyzed and plotted using R version 4.4.1 (R Core team, 2024) and RStudio (R Studio team, 2024). Data manipulation (includes filtering, summation and reshaping) was performed using dplyr version 1.1.4 (Wickham et al. 2014), tidyr version 1.3.1 (Wickham et al. 2014) and Hmisc version 5.1‐3 (Harrell 2003), also using reshape2 (Wickham 2007) and ggExtra version 0.10.1 (Attali and Baker 2015). Scatterplots and density plots were generated using ggplot2 (Wickham et al. 2007), Venn diagrams with VennDiagram version 1.7.3 (Chen 2011) and the Sankey plot using ggalluvial version 0.12.5 (Brunson and Read 2017). Area‐proportional Venn diagrams were generated using eulerr (Larsson 2016) and overlap tests were performed in R using phyper.

## 3 Results

### 3.1 CpG Methylation is Highest in Exons

**Figure 1:**
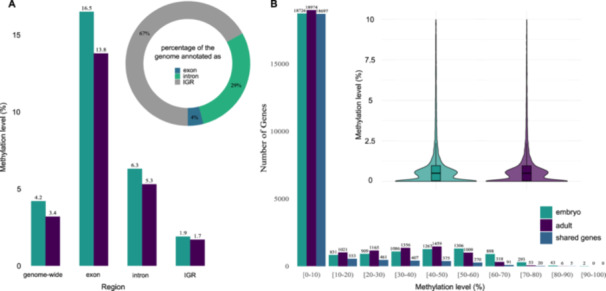
Distribution of mean CpG methylation levels. (A) Across different genomic regions and (B) Across genes, in embryos and adults. Inset in A: percentage of the genome annotated as exon, intron or intergenic region. Inset in B: zoom onto the distribution of genes in the (0–10) interval.

To quantify CpG methylation, we performed EM‐seq on three replicates each of embryo and adult samples of *L. decemlineata*. On a genome‐wide level, 4.2% and 3.4% of all CpGs were methylated in embryos and adults, respectively. Among genomic features, exons show the highest mean percent methylation with 16.5% and 13.8% in embryos and adults, respectively, followed by introns, while intergenic regions have very low levels of methylation (Figure 1A, Table S5‐6). The seemingly large number of CpGs in introns can be explained by the different total size of intron and exon sequences in the genome (Figure 1A). Of the entire *L. decemlineata* genome, approximately 67% was classified as repeats, which is similar to (Yan et al. 2023). Transposable elements (LINEs, LTR elements, DNA transposons) cover approximately 41% of the genome and have a methylation level of 3.2%. The remaining repeats are almost exclusively unclassified.

Focusing on genes, the majority of genes are not methylated (below 10%). Most of the methylated genes show mean methylation levels up to 70% while only 1.5% of genes in embryos and 0.3% in adults show methylation levels above 70% (Figure 1B).

### 3.2 Dividing Annotated Genes Into Four Subsets Based on Methylation and Expression Status

To study the relationship between gene expression, gene body methylation and histone modifications, we generated RNA‐seq data for embryo and adult samples.

Comparing embryo and adult gene expression, we identified 5,135 differentially expressed genes; 3,790 with higher expression in adults and 1,345 genes with higher expression in embryos (Figure S10, Table S7). From now on, a differentially expressed gene (DEG) will be referred to as “downregulated” if the expression is higher in the embryo compared to the adult, otherwise as “upregulated”.

Due to the pronounced level of methylation in genes, we narrowed our analysis down to a consolidated set of 25,631 protein‐coding genes. In this set, all genes had sufficient coverage of DNA methylation and RNA‐seq data for all replicates. We then assigned genes to the categories *‘not methylated’*, i.e. methylation level below 10% vs. *‘methylated’*; and *‘not expressed’*, i.e. FPKM 1 or below *vs. ‘expressed’*. In combination, this results in four groups of genes, namely, genes which are *‘not methylated/expressed’*, *‘methylated/expressed’*, *‘not methylated/not expressed’*, and *‘methylated/not expressed’*. In the following, all our analyses will refer to these four mutually exclusive gene sets if not stated otherwise. The biggest set is *‘not methylated/not expressed’* and the smallest set is *‘methylated/not expressed’*. The number of genes in each set for embryo and adult can be found in Table S8.

### 3.3 *‘Methylated/expressed*’ Genes are Usually Longer and Exclusively Characterized by a Drop in CpG Methylation at the TSS

**Figure 2:**
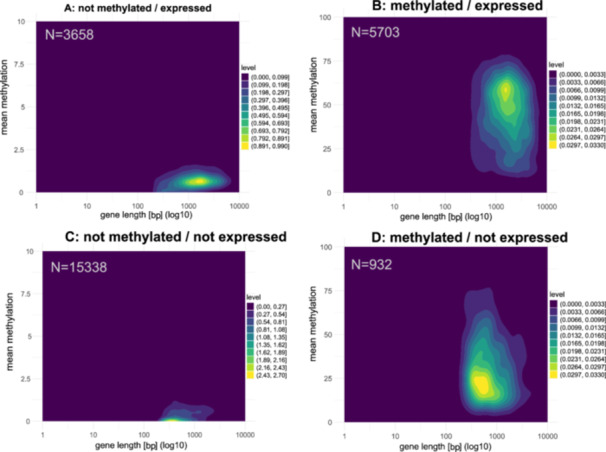
Relationship between gene length and mean methylation (%) in different categories. (Note difference in scale). The color gradient (level) represents the density of genes at different methylation levels, with brighter shades indicating regions of higher density.

We found *‘methylated/expressed’* genes in both embryo and adult exhibit the longest mean length (appr. 2 kb), while *‘not methylated/not expressed’* genes displayed the shortest mean lengths (720 and 820 bp, respectively), see Figure 2 (Figure S11 for adults). Certain subsets displayed considerable variability in their gene length, highest in *‘methylated/not expressed’* genes and lowest in *‘not methylated/not expressed’* genes, see Table S9.

**Figure 3:**
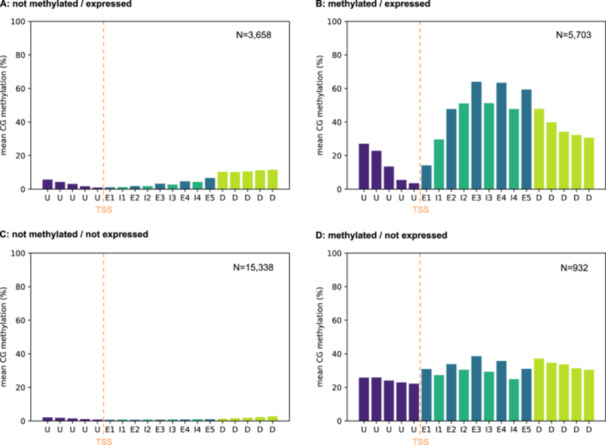
Mean embryonic methylation level of different genic segments. E1 to E5 represent the first 5 exons; I1–I4 represent the first four introns; U – upstream region; D – downstream region.

We examined the CpG methylation of the gene body as well as the 3 kb upstream and downstream regions in embryos and adults. In general, exon methylation exceeds intron methylation as described in previous studies (Lewis et al. 2020), and gene body methylation is more pronounced than methylation of non‐genic flanking regions (see Figure S12, Tables S10, S11). Surprisingly, we observe a prominent drop in CpG methylation at the TSS in *‘methylated/expressed’* genes that is clearly absent in *‘methylated/not expressed’* genes. Furthermore, CpG methylation levels in the downstream regions decrease only slowly with greater distance from the transcription end site (Figure 3).

### 3.4 Gene Body Methylation is Weakly Associated With Transcription, but Changes in GBM are not Related to Transcriptional Changes

**Figure 4:**
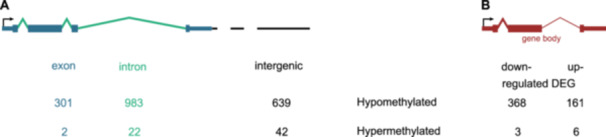
(A) Differentially methylated regions (DMRs) per genic feature. Shown are the numbers of differentially methylated regions fully contained within one genic feature using the embryo stage as reference. DMRs overlapping multiple genic features are not taken into account. (B) Relationship between differentially methylated genes and differentially expressed genes (DEGs).

We identified 3,822 significantly differentially methylated regions (DMRs) between the adult and embryo stage. 3,754 DMRs have a lower methylation in adults compared to embryos, we denote them hypomethylated. 68 DMRs are hypermethylated in the adult stage. Most hypo‐ and about 40% of the hypermethylated DMRs overlap with genes while only 17% of the hypo‐ but 61% of the hypermethylated DMRs are located in the intergenic region. Of the hypomethylated DMRs that overlap genes, 1,573 overlap only one genic feature; the same applies to 25 hypermethylated DMRs. 1,542 hypo‐ and 1 hypermethylated DMR overlap both exons and introns (see Figure 4).

**Figure 5:**
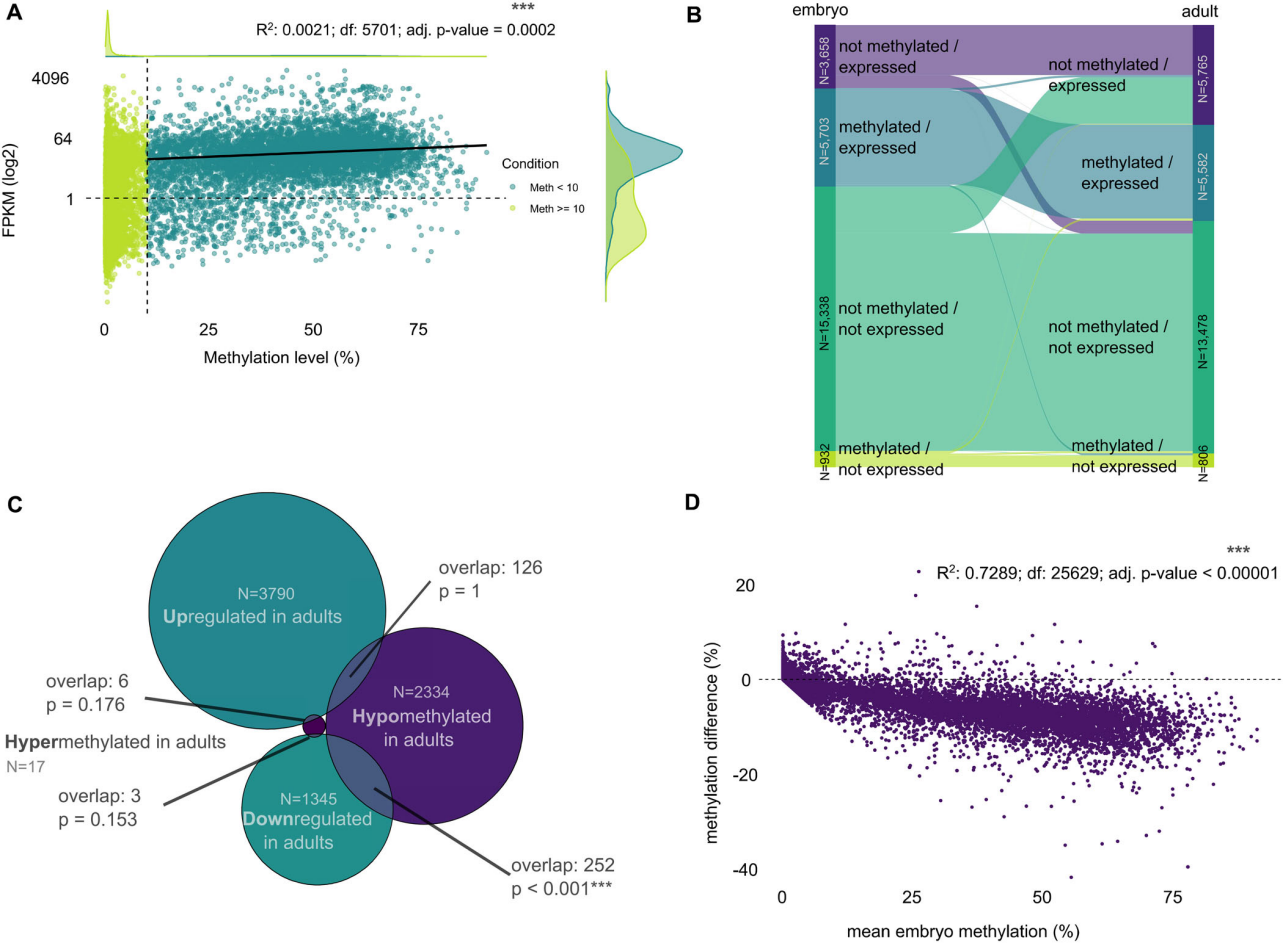
Changes in gene body methylation and gene expression. (A) Association between gene expression level (FPKM) and gene body methylation level in embryos. Dashed gray lines indicate significance thresholds. All values that equal 0 were removed and linear regression was calculated on the subset of methylated/expressed genes. (B) Sankey diagram showing the change of gene expression and methylation between embryo and adult stage. (C) Relationship of gene body methylation and gene expression between embryo and adult. Shown is an area‐proportional Venn diagram indicating both the numbers and the overlap between differentially expressed genes and differentially methylated genes of a certain direction. An overlap test was performed for all combinations, with the p‐values indicated in the figure. (D) Observed changes in gene body methylation from embryo to adult relative to embryonic methylation levels.

Exploring the association between gene body methylation and gene expression we found a weak but significant positive linear correlation in ‘*methylated/expressed’* genes for both embryos and adults (Figure 5A; Figure. S13 for adults). However, methylation explains only a small portion of the variability in expression, and neither linear nor quadratic regression models account for much of the total variation (see Figure S14). We cannot determine whether methylation promotes gene expression or v*ice versa*. A matrix summarizing ‘*methylated’* and ‘*not methylated’ genes*, categorized by their expression status (‘*expressed’/'not expressed'*), is provided in Figure S15. Moreover, methylation does not affect the strength of transcriptional variance between replicates (Figure S16), nor is there a difference in methylation levels of genes with versus without DMRs from embryo to adults (Figure S17).

We examined changes in gene expression and CpG methylation accompanying the transition from embryo to adult between the four categories (Figure 5B). 9,361 (36.5%) and 11,347 (44.3%) genes are expressed in embryos and adults, respectively. While 3,722 genes change their expression status (2,854 become active, 868 become inactive) during the transition, only 4.2% of genes lose methylation and even fewer genes (0.5%) gain methylation during this transition, while most genes (95.3%) maintain their methylation status (Tables S12‐13). However, there is a small but significant association between the transcriptional change and a change in gene body methylation. Only a very small part of the variation/change is explained by the change in GBM (Figure S18). Hypomethylation in adults is significantly associated with downregulation of the respective genes, while otherwise hyper‐ or hypomethylation is not significantly associated with directional regulation (Figure 5C). The embryonic gene body methylation level of a gene can in principle predict changes in GBM level between the developmental stages, as higher methylated genes are more likely to be less methylated in the adult stage (Figure 5D).

### 3.5 Gene Ontology Enrichment Indicates Functional Differences of Methylated and not Methylated Genes

**Figure 6:**
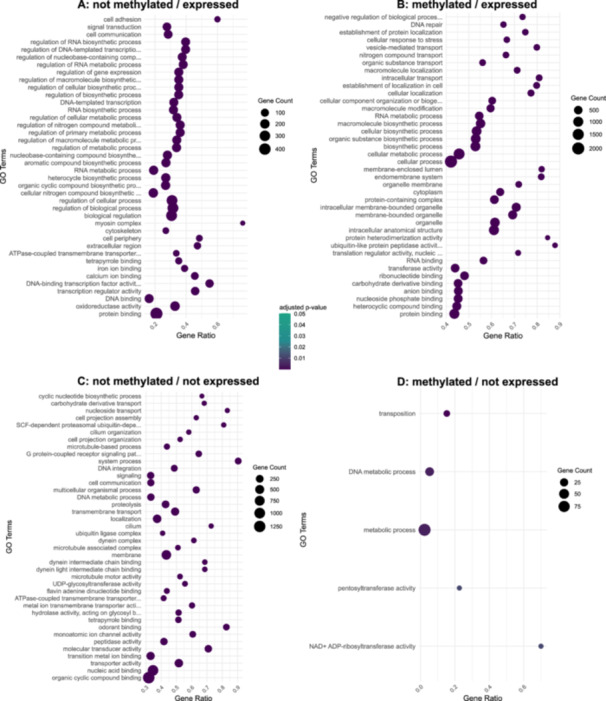
Embryo top 40 significantly enriched Gene Ontology (GO) terms for the four subsets. The *p*‐value was adjusted using the Benjamini‐Hochberg procedure. Gene count is the number of genes associated with a GO term, whereas ‘Gene Ratio’ is the percentage of genes of the specific subset in the given GO terms. Categories vary in the number of genes included, resulting in different numbers of enriched GO terms (*‘not methylated/expressed’* = 140 GO; *‘methylated/expressed*’ = 258 GO; *‘not methylated/not expressed*’ = 126 GO; ‘*methylated/not expressed*’ = 5 GO).

To further characterize which genes are expressed in the four categories, we performed a GO term analysis of the respective subsets of genes for the embryo samples. Compared to the entirety of GO terms associated with all genes, 258 GO terms were overrepresented in the ‘*methylated/expressed’* group, but only five in the ‘*methylated/not expressed’* group. However, it should be noted that this group consists of fewer genes. Among those five are GO terms related to transposition. ‘*Not methylated/expressed’* genes are frequently assigned to GO terms associated with regulatory functions, while ‘*methylated/expressed’* genes have slightly more GO terms assigned to cellular components (Figure 6).

*‘Not methylated/expressed’* genes have functional enrichment in DNA binding and transcriptional regulation as key molecular processes. Biological processes include regulation of gene expression and RNA‐related heterocycle biosynthetic pathways. ‘*Methylated/expressed’* genes show enrichment for biological processes like intracellular transport, with molecular functions focused on transmembrane transporter activity and RNA binding. Furthermore, methylation‐related genes are enriched (39 of 53 entries), chromosome segregation, TOR signaling and RNA modifications. Molecular processes highlight N‐acyltransferase activity (20 of 24 entries) and histone‐modifying activity (27 of 33 entries). In the most numerous groups of genes, ‘*not methylated/not expressed’*, the enriched GO terms include transporter activity, transmembrane transport, and cellular components like the dynein complex, indicating potentially overarching functions in cellular transport mechanisms.

Due to the high number of shared genes in the respective categories, GO terms for adult categories are overall analogous to embryos and are therefore shown in Figure S19. Examining the function of genes with more than 10% difference in their methylation levels between embryos and adults (1359 hypomethylated and 5 hypermethylated genes) reveals GO terms primarily associated with metabolic processes, binding activity, cellular localization, and organelles. Significant GO terms are shown in Figure S20. We further investigated those genes that we classified as ‘*methylated*’ in embryos but as ‘*not methylated*’ in adults. No significant GO terms could be assigned to this set of genes. We provide a table of genes and their respective GO terms from this set, in which the methylation difference is more than 10% (Table S14).

### 3.6 Histone Modifications are Strongly Associated With Active Gene Expression, and Enrichment Patterns Differ With DNA Methylation Status

We used CUT&Tag to analyze the enrichment profile of histone modifications H3K27ac and H3K36me3 in embryos. From our initial data set, we selected the 2.5% of highest peaks of each replicate and modification. We used the called peaks from both replicates to create a high confidence set of peaks by taking only the intersections of overlapping peaks into account. Out of this set of most reliable peaks, the majority are covering genes, namely 79% and 83% for H3K27ac and H3K36me3, respectively, with a remaining 21% and 17% being placed in intergenic regions.

Both H3K27ac and H3K36me3 are associated with active gene expression. 66% of ‘*expressed’* genes show an overlap with high‐confidence peaks for either H3K27ac or H3K36me3. In contrast, this is only the case for 10% of ‘*not expressed’* genes. Furthermore, 58% of expressed genes show enrichment for both modifications simultaneously, which is only the case for 4% of not expressed genes (Figure S21).

**Figure 7:**
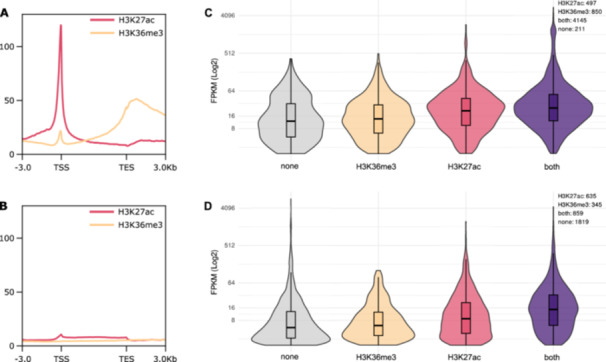
Enrichment patterns of H3K27ac and H3K36me3 in *‘expressed’* (A) and *‘not expressed’* (B) genes. Shown are the profiles for replicate 1 (replicate 2 see Figure S22). Differences in expression levels of (C) *‘methylated’* and (D) *‘not methylated’* active genes in relation to their histone modifications.

A prominent, narrow peak for H3K27ac can be seen at the transcription start site (TSS) of expressed genes (see Figure 7A). We also observe a small dip of H3K27ac levels around the transcription end site (TES). In inactive genes, only low levels of H3K27ac are observed, with a subtle peak at the TSS followed by a plateau extending to the TES (see Figure 7B).

In contrast to the tall and narrow peak of H3K27ac at the TSS, H3K36me3 presents itself with broad enrichment increasing towards the TES of the gene, followed by a gradual decline reaching far into the downstream flanking region (see Figure 7A).

The overall expression of ‘*methylated’* genes is higher, with a more pronounced effect in the presence of H3K36me3 or H3K27ac. When both modifications are present, the highest FPKM scores are observed (see Figure 7C‐D).

**Figure 8:**
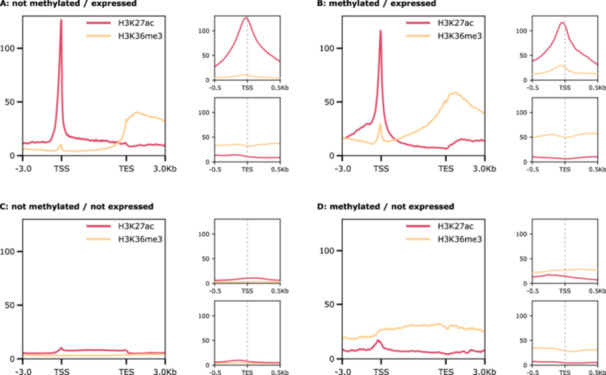
Enrichment profiles of the H3K27ac and H3K36me3 distribution at gene body, transcription start site (TSS) and transcription end site (TES). Genes are normalized to a length of 5 kb, with 3 kb upstream and downstream of TSS and TES, respectively. For TSS and TES, flanking regions of 0.5 kb were used. Figures showing the TSS and TES with larger flanking regions can be found in Figure S24. Shown are the profiles of replicate 1 (profiles for replicate 2 and heatmaps for all replicates see Figure S25).

Comparing the four categories, notable differences between ‘*not methylated*’ and ‘*methylated*’ genes can be observed in genes marked with H3K36me3. In ‘*methylated/expressed*’ genes, there is a distinct peak at the TSS, whereas in ‘*not methylated/expressed*’ genes this peak is barely visible (Figure 8A‐B). No enrichment of H3K36me3 is observed in ‘*not methylated/not expressed*’ genes, which is in stark contrast to ‘*methylated/not expressed*’ genes, where enrichment is observed over the entire gene body (Figure 8C‐D). In H3K27ac, the enrichment upstream of the TSS of ‘*methylated/expressed*’ genes is higher compared to ‘*not methylated/expressed*’ genes, while the overall enrichment pattern is similar. We performed overlap tests for both histone modifications in the four categories. For H3K27ac, overlaps are significant in ‘*not methylated/expressed*’ and ‘*methylated/expressed’* genes, but not for either of the ‘*not expressed*’ categories. In contrast, H3K36me3 shows an additional significant overlap with ‘*methylated/not expressed’* genes, suggesting a relationship between DNA methylation and H3K36me3. Area‐proportional Venn diagrams visualizing the overlaps of the histone modifications with the four gene sets and the respective p‐values are shown in Figure S23. Taken together, we observe strong enrichment of the two histone modifications in expressed genes. Unlike H3K27ac, H3K36me3 also has notable enrichment in ‘*methylated/not expressed*’ genes.

## 4 Discussion

In this study, we investigated for the first time the genome‐wide epigenetic landscape of a species that has lost the *de novo* DNA methyltransferase DNMT3 but retained the maintenance DNA methyltransferase DNMT1 and CpG methylation, the Colorado potato beetle *L. decemlineata*. Despite the loss of DNMT3, *L. decemlineata* retained CpG methylation. Genome‐wide CpG methylation levels differ between embryos and adults, and H3K36me3 enrichment reflects CpG methylation patterns, with prominent differences in expressed and non‐expressed genes, whereas H3K27ac showed a prominent peak at the transcription start of methylated and not methylated active genes.

For our multiomics approach in the Colorado potato beetle, a non‐model species in molecular biology, we applied new techniques, EM‐seq and CUT&Tag, to study CpG methylation and histone modifications, respectively. To our knowledge, it is the first time EM‐seq has been applied to an arthropod species. EM‐seq promises greater uniformity of GC coverage, and a greater coverage of CpGs at a lower required sequencing coverage depth compared to whole genome bisulfite sequencing (WGBS) (Vaisvila et al. 2021). To generate genome‐wide histone modification maps for H3K27ac and H3K36me3, we used CUT&Tag, a more sensitive alternative to ChIP‐seq, that requires a lower number of cells, making it particularly suited for small organisms like insects (Kaya‐Okur et al. 2019, 2020).

We observed genome‐wide CpG methylation levels of 4.2% in embryos and 3.4% in adults, which is rather high compared to other coleopteran species (*T. castaneum*: loss of DNMT3 and CpG methylation (Schulz et al. 2018), *N. vespilloides*: CpG methylation below 1% (Cunningham et al. 2015) and the previously reported levels in *L. decemlineata* (Brevik et al. 2021). Increased sensitivity of EM‐seq compared to WGBS (Vaisvila et al. 2021) or improved genome assembly might contribute to our higher methylation levels.

In invertebrates, unlike vertebrates, DNA methylation does not silence inactive genes (Field et al. 2004); instead, gene body methylation in insects often correlates with active transcription (Zemach et al. 2010). The fraction of methylated genes varies strongly among insect genomes. In *L. decemlineata*, around 25% of annotated genes are methylated to varying degrees, showing a nearly flat distribution between 10% and 70%, unlike the bimodal pattern seen in Hymenoptera (Dixon and Matz 2022). While gene body methylation is linked to active transcription, its regulatory role is debated. Studies suggest higher methylation correlates with increased gene expression (Foret et al. 2009; Lewis et al. 2020; Provataris et al. 2018), and we observed a positive, though small correlation, supporting the idea that gene body methylation facilitates smooth transcription rather than regulating it (de Mendoza et al. 2020; Dixon and Matz 2022). Regulatory genes require flexible expression, to which differential methylation may contribute. This has been debated in social insects, where differential DNA methylation of regulatory genes was shown between sexes and castes (Marshall et al. 2023; Okwaro and Korb 2023). However, in the non‐social beetle *L. decemlineata*, we did not observe differential methylation predominantly in regulatory genes. In absence of DNMT3, such observations would be less likely. Accordingly, we see that in both embryos and adults, regulatory genes tend to be not methylated at all, and may rely on other regulatory processes. Including additional developmental stages may give us a more detailed idea of the roles of regulatory genes and their potential differential methylation in the future.

In *L. decemlineata*, exons are more highly methylated than introns or flanking regions, with increased methylation in later exons of expressed genes, suggesting potential regulatory relevance. This aligns with an unclear trend in invertebrates, where preferentially methylated exons vary: *N. vespilloides* shows higher methylation in the first three exons, while *Blattella germanica* has elevated methylation starting from exon four (Lewis et al. 2020). Methylation drops at transcription start sites (TSS) in expressed genes, a feature absent in not expressed genes. Unmethylated promoters are essential for transcriptional initiation in vertebrates (Isagawa et al. 2011). While unmethylated promoters are common in insects, some exceptions (e.g., *Planococcus citri* and *Strigamia maritima*) show methylated promoters (Lewis et al. 2020). Reduced TSS methylation, as previously reported in the purple sea urchin *Strongylocentrotus purpuratus*, may enhance chromatin accessibility and gene expression also in our study organism (Bogan et al. 2023).

In *L. decemlineata*, ‘*methylated/expressed’* genes were, on average, the longest, which is similar to other invertebrates but contrasting to honeybee and silk moth, where highly methylated genes are shorter compared to lowly methylated genes (Sarda et al. 2012). However, these associations could be species specific and even without further regulatory relevance.

Using GO analysis, we found that *‘not methylated/expressed’* genes are often linked to regulatory processes requiring flexible expression, while ‘*methylated/expressed’* genes are associated with stable expression and roles in DNA repair and stress responses. Methylation in active genes may support stable regulation, whereas unmethylated genes allow for more dynamic expression, mediated by other transcriptional regulators. While we did not see more variation in between‐replicate expression of methylated vs not methylated genes, consistent with this, we observed that not methylated genes more frequently switch expression between embryo and adult stages compared to methylated genes. ‘*Methylated/not expressed’* genes lack a TSS dip, suggesting that methylation may suppress transcription by restricting transcription factor access, likely reducing expression of potentially harmful genes, such as those with transposase activity.

DNA methylation levels in *L. decemlineata* are low compared to other insects such as Hymenoptera. This could be attributed to the loss of *de novo* methylase DNMT3. Kucharski et al. (2008) have shown that the RNAi of DNMT3 in honey bee larvae results in queen‐like workers, therefore linking DNMT3 to caste differentiation and phenotypic plasticity. To understand the underlying mechanisms and the overall effect of DNA methylation, insects that have lost DNMT3, such as *L. decemlineata*, are of great interest. This may help to gain insights into how the loss of *de novo* methylation is compensated.

In mammals, DNMT3 plays an important role in epigenetic germline reprogramming, a process involving active and passive DNA demethylation in the zygote and during gametogenesis followed by establishment of cell‐type specific DNA methylation patterns by DNMT3 (Mayer et al. 2000; Auclair et al. 2014). This global removal is absent in insects (Yagound et al. 2020), suggesting DNMT1 alone is sufficient to maintain CpG methylation patterns in the absence of DNMT3 (Glastad et al. 2019). In the pathogenic fungus *Cryptococcus neoformans*, DNA methylation has been maintained over millions of years, despite the loss of a *de novo* methylase (Catania et al. 2020). It has been proposed that this was possible due to the high specificity of the maintenance methyltransferase DNMT5, comparable to DNMT1 in our study species. If germline methylation also persists with high fidelity over long evolutionary timescales in *L. decemlineata*, the observed differences between methylation levels of embryos and adults in our study may result from the underrepresentation of germline tissue in adult whole‐body samples.

Under certain conditions, DNMT1 may act as a *de novo* methyltransferase (Haggerty et al. 2021; Ito et al. 2024; Yarychkivska et al. 2018), potentially compensating for DNMT3 loss either directly or through interactions with other epigenetic factors. In certain insect species, like the honey bee and the locust *Locusta migratoria*, DNMT1 is duplicated, although the functional implications remain unclear (Robinson et al. 2016; Bewick et al. 2017). A possible DNMT1 duplication in *L. decemlineata* with relevance for potential *de novo* methylation warrants further investigation.

As the overarching goal of our study was to uncover how the loss of DNMT3 affects the methylation landscape and the distribution of histone modification, we studied embryonic H3K36me3 and H3K27ac patterns. In vertebrates, both histone modifications are involved in marking active genes. The interaction between H3K36me3 and methylated cytosine is deeply conserved in evolution. For this reason, H3K36me3 patterns in *D. melanogaster* (lacking a CpG methylation system) can predict the CpG methylation landscape in *S. invicta*, *A. mellifera*, and *B. mori* (Hunt et al. 2013; Nanty et al. 2011). We also find a positive association between the abundance of H3K36me3 and CpG methylation at actively expressed genes.

In vertebrates, H3K36me3 enrichment increases towards the 3'‐end of a transcribed gene and decays after the TES (Neri et al. 2017). While this is generally reflected in our data, we surprisingly observe high H3K36me3 enrichment levels extending more than 3 kb into the downstream flanking region of the gene. Our data also corroborates the enrichment of H3K36me3 at the TSS, as previously described by Zhang et al. (2022), though the level of H3K36me3 is significantly lower at the TSS compared to the TES. This peak may actually be independent of the main, genic H3K36me3 enrichment pattern, since a different enzyme, that is, SMYD5, is possibly responsible for setting this mark (Zhang et al. 2022). In contrast to not methylated and inactive genes, which show no enrichment of H3K36me3, ‘*methylated/not expressed’* display uniform H3K36me3 enrichment across the entire gene body. In general, the shapes of H3K36me3 enrichment profiles and CpG methylation levels seem to mirror each other.

If DNMT3 is the only enzyme able to methylate regions *de novo* marked by H3K36me3, through recognition by its PWWP domain, then methylation patterns observed from embryo to adult stages in *L. decemlineata* would reflect germline patterns maintained by DNMT1, with some methylation loss in somatic cells. This aligns with our findings of reduced methylation from embryonic to adult stages, though some genes appear to gain methylation during development. Further studies could explore if and how CpG methylation is acquired in *L. decemlineata*.

H3K27ac, unlike the neutral methylation marks, directly increases DNA accessibility and likely marked active genes in early eukaryotes (Prohaska et al. 2010; Iyer et al. 2008). In vertebrates, H3K27ac is found on active genes and enhancers, and our data shows a prominent TSS peak indicating active transcription. While enzymes like CBP/p300 add H3K27ac, Xu et al. (2021) suggest MBD2/3 binds to intragenic methylated CpG near the TSS, recruiting Tip60 acetyltransferase to promote H3K27ac. However, we observed that enrichment of H3K27ac is tied to transcriptional status rather than CpG methylation, challenging the generality of the previously reported link between H3K27ac and CpG methylation (Xu et al. 2021).

Histone modifications regulate transcriptional processes, and several signals need to align before transcription initiation. These modifications act as a kind of molecular memory ‐ some marks are made in advance, setting the stage for future gene activity. Similarly, when an epigenetic pattern is deconstructed, remnants can persist, reflecting past regulatory events. This might explain why we observe variance in associations of histone modifications with DNA methylation and gene expression.

## 5 Conclusion

We studied the genome‐wide CpG distribution in *L. decemlineata* using EM‐seq for the first time in an insect species. Even though this species lost DNMT3, the levels of CpG methylation were surprisingly high compared to other coleopteran species, with exons being most highly enriched in CpG methylation. Consistent with the loss of *de novo* methylation activity, we mainly observed a reduction in methylated genes in adults compared to embryos. The association between CpG methylation and H3K36me3 enrichment, which are mirroring each other, is not affected by the loss of DNMT3. H3K27ac enrichment is observed only in expressed genes, regardless of their methylation status. Taken together, our study demonstrates that connections between the different epigenetic systems show evolutionary flexibility and therefore urges caution regarding too simplistic generalizations.

## Author Contributions

Sonja J. Prohaska and Joachim Kurtz designed and supervised the study. Zoe M. Länger conducted the laboratory experiment. Zoe M. Länger., Agata I. Kalita and Claudia I. Keller Valsecchi did the CUT&Tag experiment. Zoe M. Länger, Elisa Israel, and Jan Engelhardt analyzed the data. Zoe M. Länger and Elisa Israel wrote the manuscript. Sonja J. Prohaska and Joachim Kurtz revised the manuscript. All authors read, commented, and approved the final version of the manuscript.

## Conflicts of Interest

The authors declare no conflict of interest.

## Supporting information

Laenger Israel et al supplement.

## Data Availability

All additional data supporting this article are included in the supplementary file(s). Raw sequencing data is available in the NCBI SRA database, PRJNA1179450. Reviewer Link: https://dataview.ncbi.nlm.nih.gov/object/PRJNA1179450?reviewer=oeqv2spcnmnn0gsijn5co1cc39.

## References

[jezb23303-bib-0001] Alexa, A. , and J. Rahnenfutter . 2024. topGO: Enrichment Analysis for Gene Ontology . R package version 2.59.0. 10.18129/B9.BIOC.TOPGO.

[jezb23303-bib-0002] Aliaga, B. , I. Bulla , G. Mouahid , D. Duval , and C. Grunau . 2019. “Universality of the DNA Methylation Codes in Eucaryotes.” Scientific Reports 9, no. 1: 173. 10.1038/s41598-018-37407-8. 30655579 PMC6336885

[jezb23303-bib-0003] Attali, D. , and C. Baker . 2015. “GgExtra: Add marginal histograms to ‘ggplot2’, and more ‘ggplot2’ enhancements [Dataset].” In *CRAN: Contributed Packages* . The R Foundation. 10.32614/cran.package.ggextra.

[jezb23303-bib-0004] Auclair, G. , S. Guibert , A. Bender , and M. Weber . 2014. “Ontogeny of CpG Island Methylation and Specificity of DNMT3 Methyltransferases During Embryonic Development in the Mouse.” Genome Biology 15, no. 12: 545. 10.1186/s13059-014-0545-5. 25476147 PMC4295324

[jezb23303-bib-0005] Bewick, A. J. , K. J. Vogel , A. J. Moore , and R. J. Schmitz . 2017. “Evolution of DNA Methylation Across Insects.” Molecular Biology and Evolution 34, no. 3: 654–665. 10.1093/molbev/msw264. 28025279 PMC5400375

[jezb23303-bib-0006] Bogan, S. N. , M. E. Strader , and G. E. Hofmann . 2023. “Associations Between DNA Methylation and Gene Regulation Depend on Chromatin Accessibility During Transgenerational Plasticity.” BMC Biology 21, no. 1: 149. 10.1186/s12915-023-01645-8. 37365578 PMC10294446

[jezb23303-bib-0007] Bourc'his, D. , and T. H. Bestor . 2004. “Meiotic Catastrophe and Retrotransposon Reactivation in Male Germ Cells Lacking Dnmt3L.” Nature 431, no. 7004: 96–99. 10.1038/nature02886. 15318244

[jezb23303-bib-0008] Brevik, K. , E. M. Bueno , S. McKay , S. D. Schoville , and Y. H. Chen . 2021. “Insecticide Exposure Affects Intergenerational Patterns of DNA Methylation in the Colorado Potato Beetle.” Evolutionary Applications 14, no. 3: 746–757. 10.1111/eva.13153. 33767749 PMC7980262

[jezb23303-bib-0009] Brunson, J. C. , and Q. D. Read . 2017. “ggalluvial: Alluvial Plots in ‘ggplot2’ [Dataset].” In *CRAN: Contributed Packages* . The R Foundation. 10.32614/cran.package.ggalluvial.

[jezb23303-bib-0010] Cardoso‐Júnior, C. A. M. , B. Yagound , I. Ronai , E. J. Remnant , K. Hartfelder , and B. P. Oldroyd . 2021. “DNA Methylation is not a Driver of Gene Expression Reprogramming in Young Honey Bee Workers.” Molecular Ecology 30, no. 19: 4804–4818. 10.1111/mec.16098. 34322926

[jezb23303-bib-0011] Catania, S. , P. A. Dumesic , H. Pimentel , et al. 2020. “Evolutionary Persistence of DNA Methylation for Millions of Years After Ancient Loss of a De Novo Methyltransferase.” Cell 180, no. 2: 263–277.e20. 10.1016/j.cell.2019.12.012. 31955845 PMC7197499

[jezb23303-bib-0012] Chen, H. 2011. “VennDiagram: Generate high‐resolution Venn and Euler plots [Dataset].” In CRAN: Contributed Packages. The R Foundation. 10.32614/cran.package.venndiagram.

[jezb23303-bib-0013] Cunningham, C. B. , L. Ji , R. A. W. Wiberg , et al. 2015. “The Genome and Methylome of a Beetle With Complex Social Behavior, Nicrophorus Vespilloides (Coleoptera: Silphidae).” Genome Biology and Evolution 7, no. 12: 3383–3396. 10.1093/gbe/evv194. 26454014 PMC4700941

[jezb23303-bib-0014] Dhayalan, A. , A. Rajavelu , P. Rathert , et al. 2010. “The Dnmt3a PWWP Domain Reads Histone 3 Lysine 36 Trimethylation and Guides DNA Methylation.” Journal of Biological Chemistry 285, no. 34: 26114–26120. 10.1074/jbc.M109.089433. 20547484 PMC2924014

[jezb23303-bib-0015] Dixon, G. , and M. Matz . 2022. “Changes in Gene Body Methylation do not Correlate With Changes in Gene Expression in Anthozoa or Hexapoda.” BMC Genomics 23, no. 1: 234. 10.1186/s12864-022-08474-z. 35337260 PMC8957121

[jezb23303-bib-0016] Edmunds, J. W. , L. C. Mahadevan , and A. L. Clayton . 2008. “Dynamic Histone H3 Methylation During Gene Induction: HYPB/Setd2 Mediates all H3K36 Trimethylation.” EMBO Journal 27: 406–420. 10.1038/sj.emboj.7601967. 18157086 PMC2168397

[jezb23303-bib-0017] Engelhardt, J. , O. Scheer , P. F. Stadler , and S. J. Prohaska . 2022. “Evolution of DNA Methylation Across Ecdysozoa.” Journal of Molecular Evolution 90, no. 1: 56–72. 10.1007/s00239-021-10042-0. 35089376 PMC8821070

[jezb23303-bib-0018] Field, L. M. , F. Lyko , M. Mandrioli , and G. Prantera . 2004. “DNA Methylation in Insects.” Insect Molecular Biology 13, no. 2: 109–115. 10.1111/j.0962-1075.2004.00470.x. 15056357

[jezb23303-bib-0019] Flynn, J. M. , R. Hubley , C. Goubert , et al. 2020. “RepeatModeler2 for Automated Genomic Discovery of Transposable Element Families.” Proceedings of the National Academy of Sciences 117, no. 17: 9451–9457. 10.1073/pnas.1921046117. PMC7196820 32300014

[jezb23303-bib-0020] Foret, S. , R. Kucharski , Y. Pittelkow , G. A. Lockett , and R. Maleszka . 2009. “Epigenetic Regulation of the Honey Bee Transcriptome: Unravelling the Nature of Methylated Genes.” BMC Genomics 10, no. 1: 472. 10.1186/1471-2164-10-472. 19828049 PMC2768749

[jezb23303-bib-0021] Glastad, K. M. , B. G. Hunt , and M. A. D. Goodisman . 2019. “Epigenetics in Insects: Genome Regulation and the Generation of Phenotypic Diversity.” Annual Review of Entomology 64: 185–203. 10.1146/annurev-ento-011118-111914. 30285490

[jezb23303-bib-0022] Grau‐Bové, X. , C. Navarrete , C. Chiva , et al. 2022. “A Phylogenetic and Proteomic Reconstruction of Eukaryotic Chromatin Evolution.” Nature Ecology & Evolution 6, no. 7: 1007–1023. 10.1038/s41559-022-01771-6. 35680998 PMC7613034

[jezb23303-bib-0023] Greenberg, M. V. C. , and D. Bourc'his . 2019. “The Diverse Roles of DNA Methylation in Mammalian Development and Disease.” Nature Reviews Molecular Cell Biology 20, no. 10: 590–607. 10.1038/s41580-019-0159-6. 31399642

[jezb23303-bib-0024] Guenther, M. G. , S. S. Levine , L. A. Boyer , R. Jaenisch , and R. A. Young . 2007. “A Chromatin Landmark and Transcription Initiation at Most Promoters in Human Cells.” Cell 130, no. 1: 77–88. 10.1016/j.cell.2007.05.042. 17632057 PMC3200295

[jezb23303-bib-0025] Haggerty, C. , H. Kretzmer , C. Riemenschneider , et al. 2021. “Dnmt1 has De Novo Activity Targeted to Transposable Elements.” Nature Structural & Molecular Biology 28, no. 7: 594–603. 10.1038/s41594-021-00603-8. PMC8279952 34140676

[jezb23303-bib-0026] Hahn, M. A. , X. Wu , A. X. Li , T. Hahn , and G. P. Pfeifer . 2011. “Relationship Between Gene Body DNA Methylation and Intragenic H3K9me3 and H3K36me3 Chromatin Marks.” PLoS One 6, no. 4: e18844. 10.1371/journal.pone.0018844. 21526191 PMC3079728

[jezb23303-bib-0027] Harrell, Jr., F. E. 2003. “Hmisc: Harrell Miscellaneous [Dataset].” In *CRAN: Contributed Packages* . The R Foundation. 10.32614/cran.package.hmisc.

[jezb23303-bib-0028] Harris, K. D. , J. Lloyd , K. Domb , D. Zilberman , and A. Zemach . 2019. “DNA Methylation is Maintained With High Fidelity in the Honey Bee Germline and Exhibits Global Non‐Functional Fluctuations During Somatic Development.” Epigenetics & Chromatin 12, no. 1: 62. 10.1186/s13072-019-0307-4. 31601251 PMC6786280

[jezb23303-bib-0029] Hunt, B. G. , K. M. Glastad , S. V. Yi , and M. A. D. Goodisman . 2013. “The Function of Intragenic DNA Methylation: Insights From Insect Epigenomes.” Integrative and Comparative Biology 53, no. 2: 319–328. 10.1093/icb/ict003. 23509238

[jezb23303-bib-0030] Isagawa, T. , G. Nagae , N. Shiraki , et al. 2011. “DNA Methylation Profiling of Embryonic Stem Cell Differentiation Into the Three Germ Layers.” PLoS One 6, no. 10: e26052. 10.1371/journal.pone.0026052. 22016810 PMC3189249

[jezb23303-bib-0031] Ito, T. , M. Kubiura‐Ichimaru , F. Miura , et al. 2024. “DNMT1 Can Induce Primary Germ Layer Differentiation Through De Novo DNA Methylation.” Genes to Cells: Devoted to Molecular & Cellular Mechanisms 29, no. 7: 549–566. 10.1111/gtc.13130. 38811355 PMC11447926

[jezb23303-bib-0032] Iyer, L. M. , V. Anantharaman , M. Y. Wolf , and L. Aravind . 2008. “Comparative Genomics of Transcription Factors and Chromatin Proteins in Parasitic Protists and Other Eukaryotes.” International Journal for Parasitology 38, no. 1: 1–31. 10.1016/j.ijpara.2007.07.018. 17949725

[jezb23303-bib-0033] Jühling, F. , H. Kretzmer , S. H. Bernhart , C. Otto , P. F. Stadler , and S. Hoffmann . 2016. “Metilene: Fast and Sensitive Calling of Differentially Methylated Regions From Bisulfite Sequencing Data.” Genome Research 26, no. 2: 256–262. 10.1101/gr.196394.115. 26631489 PMC4728377

[jezb23303-bib-0034] Kang, Y. , Y. W. Kim , J. Kang , and A. Kim . 2021. “Histone H3K4me1 and H3K27ac Play Roles in Nucleosome Eviction and eRNA Transcription, Respectively, at Enhancers.” FASEB Journal 35, no. 8: e21781. 10.1096/fj.202100488R. 34309923

[jezb23303-bib-0035] Kaya‐Okur, H. S. , D. H. Janssens , J. G. Henikoff , K. Ahmad , and S. Henikoff . 2020. “Efficient Low‐Cost Chromatin Profiling With CUT&Tag.” Nature Protocols 15, no. 10: 3264–3283. 10.1038/s41596-020-0373-x. 32913232 PMC8318778

[jezb23303-bib-0036] Kaya‐Okur, H. S. , S. J. Wu , and C. A. Codomo , et al. 2019. “CUT&Tag for Efficient Epigenomic Profiling of Small Samples and Single Cells.” Nature Communications 10, no. 1: 1930. 10.1038/s41467-019-09982-5. PMC6488672 31036827

[jezb23303-bib-0037] Krueger, F. , and S. R. Andrews . 2011. “Bismark: A Flexible Aligner and Methylation Caller for Bisulfite‐Seq Applications.” Bioinformatics 27, no. 11: 1571–1572. 10.1093/bioinformatics/btr167. 21493656 PMC3102221

[jezb23303-bib-0038] Krueger, F. , F. James , P. Ewels , et al. (2023). *FelixKrueger/TrimGalore: v0.6.10 ‐ Add Default Decompression Path*. Zenodo. 10.5281/ZENODO.7598955.

[jezb23303-bib-0039] Kucharski, R. , J. Maleszka , S. Foret , and R. Maleszka . 2008. “Nutritional Control of Reproductive Status in Honeybees via DNA Methylation.” Science 319, no. 5871: 1827–1830. 10.1126/science.1153069. 18339900

[jezb23303-bib-0040] Langmead, B. , and S. L. Salzberg . 2012. “Fast Gapped‐Read Alignment With Bowtie 2.” Nature Methods 9, no. 4: 357–359. 10.1038/nmeth.1923. 22388286 PMC3322381

[jezb23303-bib-0041] Larsson, J. 2016. “Eulerr: Area‐Proportional Euler and Venn diagrams with ellipses [Dataset].” In *CRAN: Contributed Packages* . The R Foundation. 10.32614/cran.package.eulerr.

[jezb23303-bib-0042] Lewis, S. H. , L. Ross , S. A. Bain , et al. 2020. “Widespread Conservation and Lineage‐Specific Diversification of Genome‐Wide DNA Methylation Patterns Across Arthropods.” PLoS Genetics 16, no. 6: e1008864. 10.1371/journal.pgen.1008864. 32584820 PMC7343188

[jezb23303-bib-0043] Lister, R. , M. Pelizzola , R. H. Dowen , et al. 2009. “Human DNA Methylomes at Base Resolution Show Widespread Epigenomic Differences.” Nature 462, no. 7271: 315–322. 10.1038/nature08514. 19829295 PMC2857523

[jezb23303-bib-0044] Love, M. I. , W. Huber , and S. Anders . 2014. “Moderated Estimation of Fold Change and Dispersion for RNA‐Seq Data With Deseq. 2.” Genome Biology 15, no. 12: 550. 10.1186/s13059-014-0550-8. 25516281 PMC4302049

[jezb23303-bib-0045] Lyko, F. 2017. “The DNA Methyltransferase Family: A Versatile Toolkit for Epigenetic Regulation.” Nature Reviews Genetics 19, no. 2: 81–92. 10.1038/nrg.2017.80. 29033456

[jezb23303-bib-0046] Maleszka, R. 2024. “Reminiscences on the Honeybee Genome Project and the Rise of Epigenetic Concepts in Insect Science.” Insect Molecular Biology 33, no. 5: 444–456. 10.1111/imb.12888. 38196200

[jezb23303-bib-0047] Marshall, H. , M. T. Nicholas , J. S. van Zweden , et al. 2023. “DNA Methylation is Associated With Codon Degeneracy in a Species of Bumblebee.” Heredity 130, no. 4: 188–195. 10.1038/s41437-023-00591-z. 36658299 PMC10076500

[jezb23303-bib-0048] Martin, M. 2011. “Cutadapt Removes Adapter Sequences From High‐Throughput Sequencing Reads.” EMBnet.journal 17, no. 1: 10–12. 10.14806/ej.17.1.200.

[jezb23303-bib-0049] Mayer, W. , A. Niveleau , J. Walter , R. Fundele , and T. Haaf . 2000. “Demethylation of the Zygotic Paternal Genome.” Nature 403, no. 6769: 501–502. 10.1038/35000656. 10676950

[jezb23303-bib-0050] Meers, M. P. , D. Tenenbaum , and S. Henikoff . 2019. “Peak Calling by Sparse Enrichment Analysis for CUT&RUN Chromatin Profiling.” Epigenetics & Chromatin 12, no. 1: 42. 10.1186/s13072-019-0287-4. 31300027 PMC6624997

[jezb23303-bib-0051] de Mendoza, A. , R. Lister , and O. Bogdanovic . 2020. “Evolution of DNA Methylome Diversity in Eukaryotes.” Journal of Molecular Biology 432, no. 6: 1687–1705. 10.1016/j.jmb.2019.11.003. 31726061

[jezb23303-bib-0052] Mortazavi, A. , B. A. Williams , K. McCue , L. Schaeffer , and B. Wold . 2008. “Mapping and Quantifying Mammalian Transcriptomes by RNA‐Seq.” Nature Methods 5, no. 7: 621–628. 10.1038/nmeth.1226. 18516045 PMC13303166

[jezb23303-bib-0053] Nanty, L. , G. Carbajosa , G. A. Heap , et al. 2011. “Comparative Methylomics Reveals Gene‐Body H3K36me3 in Drosophila Predicts DNA Methylation and CpG Landscapes in Other Invertebrates.” Genome Research 21, no. 11: 1841–1850. 10.1101/gr.121640.111. 21940836 PMC3205569

[jezb23303-bib-0054] Nègre, N. , C. D. Brown , L. Ma , et al. 2011. “A Cis‐Regulatory Map of the Drosophila Genome.” Nature 471, no. 7339: 527–531. 10.1038/nature09990. 21430782 PMC3179250

[jezb23303-bib-0055] Neri, F. , S. Rapelli , A. Krepelova , et al. 2017. “Intragenic DNA Methylation Prevents Spurious Transcription Initiation.” Nature 543, no. 7643: 72–77. 10.1038/nature21373. 28225755

[jezb23303-bib-0056] Okwaro, L. A. , and J. Korb . 2023. “Epigenetic Regulation and Division of Labor in Social Insects.” Current Opinion in Insect Science 58: 101051. 10.1016/j.cois.2023.101051. 37164259

[jezb23303-bib-0057] Oldroyd, B. P. , and B. Yagound . 2021. “The Role of Epigenetics, Particularly DNA Methylation, in the Evolution of Caste in Insect Societies.” Philosophical Transactions of the Royal Society, B: Biological Sciences 376: 20200115. 10.1098/rstb.2020.0115. PMC8059649 33866805

[jezb23303-bib-0058] Pokholok, D. K. , C. T. Harbison , S. Levine , et al. 2005. “Genome‐Wide Map of Nucleosome Acetylation and Methylation in Yeast.” Cell 122, no. 4: 517–527. 10.1016/j.cell.2005.06.026. 16122420

[jezb23303-bib-0059] Prohaska, S. J. , P. F. Stadler , and D. C. Krakauer . 2010. “Innovation in Gene Regulation: The Case of Chromatin Computation.” Journal of Theoretical Biology 265, no. 1: 27–44. 10.1016/j.jtbi.2010.03.011. 20303358

[jezb23303-bib-0060] Provataris, P. , K. Meusemann , O. Niehuis , S. Grath , and B. Misof . 2018. “Signatures of DNA Methylation Across Insects Suggest Reduced DNA Methylation Levels In Holometabola.” Genome Biology and Evolution 10, no. 4: 1185–1197. 10.1093/gbe/evy066. 29697817 PMC5915941

[jezb23303-bib-0061] Quinlan, A. R. , and I. M. Hall . 2010. “Bedtools: A Flexible Suite of Utilities for Comparing Genomic Features.” Bioinformatics 26, no. 6: 841–842. 10.1093/bioinformatics/btq033. 20110278 PMC2832824

[jezb23303-bib-0062] Raddatz, G. , P. M. Guzzardo , N. Olova , et al. 2013. “Dnmt2‐dependent Methylomes Lack Defined DNA Methylation Patterns.” Proceedings of the National Academy of Sciences 110, no. 21: 8627–8631. 10.1073/pnas.1306723110. PMC3666705 23641003

[jezb23303-bib-0063] Ramírez, F. , D. P. Ryan , B. Grüning , et al. 2016. “deepTools2: a Next Generation Web Server for Deep‐Sequencing Data Analysis.” Nucleic Acids Research 44, no. W1: W160–W165. 10.1093/nar/gkw257. 27079975 PMC4987876

[jezb23303-bib-0064] Robinson, K. L. , D. Tohidi‐Esfahani , F. Ponton , S. J. Simpson , G. A. Sword , and N. Lo . 2016. “Alternative Migratory Locust Phenotypes Are Associated With Differences In the Expression of Genes Encoding the Methylation Machinery.” Insect Molecular Biology 25, no. 2: 105–115. 10.1111/imb.12203. 26612460

[jezb23303-bib-0065] Sarda, S. , J. Zeng , B. G. Hunt , and S. V. Yi . 2012. “The Evolution of Invertebrate Gene Body Methylation.” Molecular Biology and Evolution 29, no. 8: 1907–1916. 10.1093/molbev/mss062. 22328716

[jezb23303-bib-0066] Schulz, N. K. E. , C. I. Wagner , J. Ebeling , et al. 2018. “Dnmt1 Has An Essential Function Despite the Absence of CpG DNA Methylation In the Red Flour Beetle *Tribolium castaneum* .” Scientific Reports 8, no. 1: 16462. 10.1038/s41598-018-34701-3. 30405203 PMC6220294

[jezb23303-bib-0067] Simola, D. F. , L. Wissler , G. Donahue , et al. 2013. “Social Insect Genomes Exhibit Dramatic Evolution In Gene Composition and Regulation While Preserving Regulatory Features Linked to Sociality.” Genome Research 23, no. 8: 1235–1247. 10.1101/gr.155408.113. 23636946 PMC3730098

[jezb23303-bib-0068] Suzuki, M. M. , A. R. W. Kerr , D. De Sousa , and A. Bird . 2007. “Cpg Methylation Is Targeted to Transcription Units In An Invertebrate Genome.” Genome Research 17, no. 5: 625–631. 10.1101/gr.6163007. 17420183 PMC1855171

[jezb23303-bib-0069] Teissandier, A. , and D. Bourc'his . 2017. “Gene Body DNA Methylation Conspires With H3K36me3 to Preclude Aberrant Transcription.” The EMBO Journal 36, no. 11: 1471–1473. 10.15252/embj.201796812. 28442531 PMC5452023

[jezb23303-bib-0070] Tie, F. , R. Banerjee , C. A. Stratton , et al. 2009. “Cbp‐Mediated Acetylation of Histone H3 Lysine 27 Antagonizes Drosophila Polycomb Silencing.” Development 136, no. 18: 3131–3141. 10.1242/dev.037127. 19700617 PMC2730368

[jezb23303-bib-0071] Vaisvila, R. , V. K. C. Ponnaluri , Z. Sun , et al. 2021. “Enzymatic Methyl Sequencing Detects DNA Methylation At Single‐Base Resolution From Picograms of DNA.” Genome Research 31, no. 7: 1280–1289. 10.1101/gr.266551.120. 34140313 PMC8256858

[jezb23303-bib-0072] Wagner, E. J. , and P. B. Carpenter . 2012. “Understanding the Language of Lys36 Methylation At Histone H3.” Nature Reviews Molecular Cell Biology 13, no. 2: 115–126. 10.1038/nrm3274. 22266761 PMC3969746

[jezb23303-bib-0073] Wickham, H. 2007. “Reshaping Data With Thereshapepackage.” Journal of Statistical Software 21, no. 12: 1. 10.18637/jss.v021.i12.

[jezb23303-bib-0074] Wickham, H. , W. Chang , L. Henry , et al. 2007. “Ggplot2: Create Elegant Data Visualisations Using the Grammar of Graphics [Dataset].” In *CRAN: Contributed Packages* . The R Foundation. 10.32614/cran.package.ggplot2.

[jezb23303-bib-0075] Wickham, H. , R. François , L. Henry , K. Müller , and D. Vaughan . 2014. “dplyr: A Grammar of Data Manipulation [Dataset].” In *CRAN: Contributed Packages* . The R Foundation. 10.32614/cran.package.dplyr.

[jezb23303-bib-0076] Wickham, H. , D. Vaughan , and M. Girlich . 2014. “tidyr: Tidy Messy Data [Dataset].” In *CRAN: Contributed Packages* . The R Foundation. 10.32614/cran.package.tidyr.

[jezb23303-bib-0077] Wilhelm, L. , Y. Wang , and S. Xu . 2025. “Gene Expression Atlas of the Colorado Potato Beetle (*Leptinotarsa decemlineata*).” Scientific Data 12, no. 1: 299. 10.1038/s41597-025-04607-7. 39971983 PMC11840028

[jezb23303-bib-0078] Xu, G. , H. Lyu , Y. Yi , et al. 2021. “Intragenic DNA Methylation Regulates Insect Gene Expression and Reproduction Through the MBD/Tip60 Complex.” iScience 24, no. 2: 102040. 10.1016/j.isci.2021.102040. 33521602 PMC7820559

[jezb23303-bib-0079] Yagound, B. , E. J. Remnant , G. Buchmann , and B. P. Oldroyd . 2020. “Intergenerational Transfer of DNA Methylation Marks In the Honey Bee.” Proceedings of the National Academy of Sciences 117, no. 51: 32519–32527. 10.1073/pnas.2017094117. PMC7768778 33257552

[jezb23303-bib-0080] Yan, J. , C. Zhang , M. Zhang , et al. 2023. “Chromosome‐Level Genome Assembly of the Colorado Potato Beetle, *Leptinotarsa decemlineata* .” Scientific Data 10, no. 1: 36. 10.1038/s41597-023-01950-5. 36653371 PMC9849343

[jezb23303-bib-0081] Yano, S. , T. Ishiuchi , S. Abe , et al. 2022. “Histone H3K36me2 and H3K36me3 Form a Chromatin Platform Essential for DNMT3A‐Dependent DNA Methylation In Mouse Oocytes.” Nature Communications 13, no. 1: 4440. 10.1038/s41467-022-32141-2. PMC9349174 35922445

[jezb23303-bib-0082] Yarychkivska, O. , Z. Shahabuddin , N. Comfort , M. Boulard , and T. H. Bestor . 2018. “Bah Domains and a Histone‐Like Motif In DNA Methyltransferase 1 (DNMT1) Regulate De Novo and Maintenance Methylation In Vivo.” Journal of Biological Chemistry 293, no. 50: 19466–19475. 10.1074/jbc.RA118.004612. 30341171 PMC6302165

[jezb23303-bib-0083] Yoh, S. M. , J. S. Lucas , and K. A. Jones . 2008. “The Iws1:Spt6:CTD Complex Controls Cotranscriptional mRNA Biosynthesis and HYPB/Setd2‐Mediated Histone H3K36 Methylation.” Genes & Development 22, no. 24: 3422–3434. 10.1101/gad.1720008. 19141475 PMC2607075

[jezb23303-bib-0084] Zemach, A. , I. E. McDaniel , P. Silva , and D. Zilberman . 2010. “Genome‐Wide Evolutionary Analysis of Eukaryotic DNA Methylation.” Science 328, no. 5980: 916–919. 10.1126/science.1186366. 20395474

[jezb23303-bib-0085] Zhang, Y. , Y. Fang , Y. Tang , et al. 2022. “SMYD5 Catalyzes Histone H3 Lysine 36 Trimethylation at Promoters.” Nature Communications 13, no. 1: 3190. 10.1038/s41467-022-30940-1. PMC9184575 35680905

[jezb23303-bib-0086] Zheng, Y. , K. Ahmad , and S. Henikoff . 2020. “CUT&Tag Data Processing and Analysis Tutorial v1 [Dataset].” In *protocols.io* . ZappyLab, Inc. 10.17504/protocols.io.bjk2kkye.

