# Supplementary material for: Multiomics Reveal Associations Between CpG Methylation, Histone Modifications and Transcription in a Species That has Lost DNMT3, the Colorado Potato Beetle
Source: J Exp Zool B Mol Dev Evol. 2025 May 12;344(7):454–69. doi: 10.1002/jez.b.23303 (PMC12576374; doi:10.1002/jez.b.23303)
Supplement: Supplementary file 1 — Laenger Israel et al supplement. [file JEZ-344-454-s001.docx]

## Supplementary methods

**Model organism and samples**

We reared Colorado potato beetles, *Leptinotarsa decemlineata* (Coleoptera), in non-overlapping generations on approximately 6-week-old potato (*Solanum tuberosum)* plants (Annabelle variety, purchased from Ellenberg´s Kartoffelvielfalt GmbH & Co. KG, Barum, Germany) in mesh cages (47.5 x 47.5 x 47.5 cm, BugDorm-4E4545, Megaview, Taichung, Taiwan) under constant conditions (16:8 light dark cycle, 70% humidity, 24 °C). Plants were grown in plastic trays in ‘Topfsubstrat D400’, Stender, in a greenhouse (16:8 light dark cycle) at 24 °C. All life stages were allowed to feed on the plant freely.

**DNA methylation: Enzymatic methyl sequencing (EM-seq)**

We submerged flash-frozen *L. decemlineata* (embryo/adult) in 195 µL TNES buffer (400mM NaCl, 200mM EDTA, 50 mM Tris pH 8.0, 0.5 % SDS) with 5 µL proteinase K (20 mg/mL) and homogenized with a sterile pestle. The homogenate was incubated at 55°C for 1h. After centrifugation (18000 g, 5min), the supernatant was taken, and an equal volume of 24:1 chloroform:isoamyl was added. We mixed by inversion and centrifuged at 10000 x g for 2 min. The aqueous phase was transferred and 60 µL NaCl and 290 µL 96 % Ethanol were added and mixed by inversion. The solution was then stored at -20 °C for 1 h and centrifuged (18000 x g, 15min) afterwards. We removed the supernatant and added 1 mL 70 % chilled Ethanol before centrifuging at 18000 x g for 5 min. Washing was repeated once, then the supernatant was removed and the pellet was left for air dry. 40 µL elution buffer (Tris-HCl pH 8.5) was added. To resuspend the pellet, we heated it to 50 °C for 3min. 2µL RNAse A was added and it was incubated at 37 °C for 30 min. DNA was cleaned before library preparation with the DNeasy PowerClean Kit, Qiagen, following the manufacturer's instructions.

**Gene expression: RNA extraction and RNA-seq**

We used a protocol combining Trizol lysis and chloroform extraction with the purification via spin columns from the SV Total RNA Isolation System (Promega). Here, 500 µl of Trizol was added to the frozen sample, which was then homogenized with a clean, sterile pestle. Another 500 µl of Trizol was added to the homogenized sample. The samples were sonicated for 10 minutes and incubated for another 10 minutes while being regularly vortexed. After a 5 min centrifugation (13000 rpm, 4 °C), the supernatant was transferred to a new 1.5 ml Eppendorf tube. 200 µl chloroform was added to the supernatant and the mix was incubated for 15 minutes under regular inversion and centrifuged afterward at 10500 rcf, 15 min, 4 °C). Following the manual instruction, the liquid phase was taken to continue with the purification via spin column.

## Supplementary tables

**Table S1. Enzymatic methyl sequencing.** The number of reads, percentage of uniquely mapped reads and coverage of the 3 embryo and 3 adult *L. decemlineata* replicates.

|  | **reads** | **% uniquely mapped (after deduplication)** | **% uniquely mapped (average after deduplication)** | **coverage** | **coverage (average)** |
| --- | --- | --- | --- | --- | --- |
| **Adult 1** | 64 mio | 52.6 | 50.2 | 8.5X | 8.7X |
| **Adult 2** | 61 mio | 52.2 |  | 8.1X |  |
| **Adult 3** | 81 mio | 45.7 |  | 9.5X |  |
| **Embryo 1** | 88 mio | 44.5 | 42.4 | 9.9X | 8.3X |
| **Embryo 2** | 81 mio | 44.6 |  | 9.2X |  |
| **Embryo 3** | 60 mio | 38.3 |  | 5.8X |  |

**Table S2. Proportions of methylated cytosines in the DNA controls.** Lambda is unmethylated DNA while pUC19 is CpG-methylated DNA. Columns 3 and 5 show the proportion (%mC) of unconverted, i.e. methylated, cytosines in the two DNA controls. If the conversion would be perfect, i.e. 100%, the Lambda DNA would not contain any C’s anymore, therefore the conversion efficiency (column 4) is calculated by subtracting ‘%mC Lambda’ from 100. The values in column 5 indicate the relative number of “over-conversion”, i.e. methylated cytosines that have been incorrectly converted to T’s and would therefore be classified as unmethylated cytosine.

| **Sample ID** | **Sample Name** | **%mC Lambda** | **%Conversion eff.** | **%mC pUC19** |
| --- | --- | --- | --- | --- |
| 215076 | Adult 1 | 0.56 | 99.44 | 91.09 |
| 215079 | Adult 2 | 0.68 | 99.23 | 89.23 |
| 215081 | Adult 3 | 0.45 | 99.55 | 89.46 |
| 215083 | Embryo 1 | 0.43 | 99.57 | 92.89 |
| 215085 | Embryo 2 | 0.32 | 99.68 | 92.47 |
| 215087 | Embryo 3 | 0.43 | 99.57 | 97.68 |
|  | **Average** | 0.48 | 99.51 | 92.14 |

**Table S3. Number of reads and percentage of uniquely mapped reads of three adult and the two remaining embryo *L. decemlineata* RNA-seq replicates.**

|  | **reads** | **% uniquely mapped** |
| --- | --- | --- |
| **Adult 1** | 62 mio | 76 |
| **Adult 2** | 75 mio | 60 |
| **Adult 3** | 68 mio | 70 |
| **Embryo 1** | removed | removed |
| **Embryo 2** | 64 mio | 75 |
| **Embryo 3** | 69 mio | 78 |

**Table S4. Number of reads and percentage of fragments mapped to the reference genome for CUT&Tag replicates.**

|  | **reads** | **paired reads (%)** | **mapped reads (%)** |
| --- | --- | --- | --- |
| **H3K27ac replicate 1** | 20568774 | 100 | 91.25 |
| **H3K27ac replicate 2** | 14155082 | 100 | 89.06 |
| **H3K36me3 replicate 1** | 20187579 | 100 | 88.05 |
| **H3K36me3 replicate 2** | 19151354 | 100 | 80.28 |

**Table S5. Distribution of CpGs to different genomic features.** The last column indicates the total amount of CpGs in the respective conditions. Columns 2-6 indicate how many CpGs are covered by the respective feature. The percent value is calculated in respect to the total number.

|  | **Exon** | **Intron** | **5’UTR** | **3’ UTR** | **IGR** | **Total** |
| --- | --- | --- | --- | --- | --- | --- |
| **Methylation ≥ 10%** | | | | | | |
| **Embryo** | 198,430  (20.7%) | 470,742  (49.1%) | 1,119  (0.1%) | 24,847  (2.6%) | 262,582  (27.4%) | 959,479 |
| **Adult** | 205,505  (20.9%) | 483,471  (49.1%) | 1,101  (0.1%) | 25,471  (2.6%) | 266,406  (27.1%) | 984,043 |
| **Methylation ≥ 90%** | | | | | | |
| **Embryo** | 26,969  (38.7%) | 29,850  (42.8%) | 161  (0.3%) | 2,593  (3.7%) | 11,807  (16.9%) | 69,747 |
| **Adult** | 7,798  (41.4%) | 7,749  (41.2%) | 50  (0.3%) | 656  (3.5%) | 3,097  (16.5%) | 18,826 |

**Table S6. Distribution of genomic features across the entire genome according to the gene annotation.**

|  | **Exon** | **Intron** | **IGR** | **Total** |
| --- | --- | --- | --- | --- |
| **Length in nt** | 37,881,039  (4%) | 271,949,241  (29%) | 638,956,388  (67%) | 948,786,668 |

**Table S7. Average log2 fold change**

|  | **average log2 fold change** | **maximum** |
| --- | --- | --- |
| **embryo upregulated** | 6.6 | 28.8 |
| **embryo downregulated** | 3.4 | 16.2 |

**Table S8. Number of genes in each subset for embryo and adult.** For all data involving **DNA methylation** and other -omics data**,** we use a set of 25,631 genes (all genes with “NA” in any of the replicates are removed, i.e. 4,988 genes of all genes in the *L. decemlineata* annotation). For embryo and adult samples, this set can be divided up into four subsets each.

|  | **Consolidated gene set** | **embryo** | **adult** |
| --- | --- | --- | --- |
| **Meth<10 AND FPKM≥1** | *‘not methylated / expressed‘* | 3,658 | 5,765 |
| **Meth≥10 AND FPKM≥1** | *‘methylated / expressed’* | 5,703 | 5,582 |
| **Meth<10 AND FPKM<1** | *‘not methylated / not expressed’* | 15,338 | 13,478 |
| **Meth≥10 AND FPKM<1** | *‘methylated / not expressed’* | 932 | 806 |
| **total** |  | 25,631 | 25,631 |

**Table S9. Gene length with standard error in the four categories for embryo and adult.**

| **Category** | **Mean gene length** | **Median gene length** | **Standard Error (SE)** |
| --- | --- | --- | --- |
| **Embryo** | | | |
| ***‘Not methylated / expressed’*** | 1903.68 | 1421.5 | 30.22 |
| ***‘Methylated /  not expressed’*** | 969.95 | 637.5 | 31.06 |
| ***‘Methylated / expressed’*** | 2075.21 | 1647.0 | 21.65 |
| ***‘Not methylated /  not expressed’*** | 822.51 | 531.0 | 7.79 |
| **Adult** | | | |
| ***‘Not methylated / expressed’*** | 1792.85 | 1369.0 | 23.18 |
| ***‘Methylated /  not expressed’*** | 910.60 | 603.0 | 32.96 |
| ***‘Methylated /expressed’*** | 2070.68 | 1646.0 | 21.75 |
| ***‘Not methylated /  not expressed’*** | 718.95 | 489.0 | 6.73 |

**Table S10. Number of regions for the different categories in the embryo barplots.**

|  | **All** | ***‘methylated / expressed’*** | ***‘methylated /***  ***not expressed’*** | ***‘not methylated / expressed’*** | ***‘not methylated / not expressed’*** |
| --- | --- | --- | --- | --- | --- |
| **UP5** | 21,569 | 5,050 | 741 | 3,130 | 12,648 |
| **UP4** | 21,720 | 5,123 | 765 | 3,128 | 12,704 |
| **UP3** | 21,981 | 5,166 | 759 | 3,124 | 12,932 |
| **UP2** | 22,387 | 5,226 | 780 | 3,186 | 13,195 |
| **UP1** | 23,250 | 5,337 | 818 | 3,373 | 13,722 |
| **E1** | 20,968 | 4,674 | 776 | 2,921 | 12,597 |
| **I1** | 13,270 | 3,413 | 259 | 3,081 | 6,517 |
| **E2** | 13,479 | 4,435 | 281 | 2,758 | 6,005 |
| **I2** | 9,824 | 3,520 | 151 | 2,568 | 3,585 |
| **E3** | 9,512 | 3,843 | 143 | 2,331 | 3,195 |
| **I3** | 7,410 | 2,957 | 94 | 2,159 | 2,200 |
| **E4** | 7,273 | 3,213 | 76 | 1,993 | 1,991 |
| **I4** | 5,894 | 2,425 | 52 | 1,786 | 1,631 |
| **E5** | 5,782 | 2,631 | 47 | 1,646 | 1,458 |
| **D1** | 22,913 | 5,248 | 780 | 3,335 | 13,550 |
| **D2** | 22,134 | 5,153 | 769 | 3,230 | 12,982 |
| **D3** | 21,742 | 5,074 | 748 | 3,182 | 12,738 |
| **D4** | 21,539 | 5,059 | 736 | 3,147 | 12,597 |
| **D5** | 21,470 | 5,001 | 738 | 3,157 | 12,574 |

**Table S11. Number of regions for the different categories in the adult barplots.**

|  | **All** | ***‘methylated / expressed’*** | ***‘methylated /***  ***not expressed’*** | ***‘not methylated / expressed’*** | ***‘not methylated / not expressed’*** |
| --- | --- | --- | --- | --- | --- |
| **UP5** | 21,908 | 4,978 | 659 | 4,960 | 11,311 |
| **UP4** | 22,055 | 5,041 | 664 | 4,982 | 11,368 |
| **UP3** | 22,288 | 5,061 | 671 | 4,994 | 11,562 |
| **UP2** | 22,691 | 5,127 | 682 | 5,136 | 11,746 |
| **UP1** | 23,515 | 5,218 | 704 | 5,387 | 12,206 |
| **E1** | 21,483 | 4,649 | 675 | 4,771 | 11,388 |
| **I1** | 13,527 | 3,360 | 211 | 4,784 | 5,172 |
| **E2** | 13,945 | 4,402 | 218 | 4,513 | 4,812 |
| **I2** | 9,939 | 3,477 | 115 | 3,961 | 2,386 |
| **E3** | 9,928 | 3,893 | 109 | 3,761 | 2,165 |
| **I3** | 7,539 | 2,953 | 59 | 3,313 | 1,214 |
| **E4** | 7,575 | 3,263 | 43 | 3,152 | 1,117 |
| **I4** | 6,013 | 2,437 | 28 | 2,700 | 848 |
| **E5** | 6,043 | 2,651 | 22 | 2,610 | 760 |
| **D1** | 23,318 | 5,212 | 684 | 5,324 | 12,098 |
| **D2** | 22,580 | 5,099 | 673 | 5,117 | 11,691 |
| **D3** | 22,119 | 5,024 | 657 | 5,026 | 11,412 |
| **D4** | 21,888 | 4,982 | 661 | 4,973 | 11,272 |
| **D5** | 21,856 | 4,951 | 636 | 4,965 | 11,304 |

**Table S12. Number of genes changing category assignment from embryo to adult.**

| embryo/adult | ***‘not methylated / expressed’*** | ***‘methylated / expressed’*** | ***‘not methylated / not expressed’*** | ***‘methylated /  not expressed’*** |  |
| --- | --- | --- | --- | --- | --- |
| ***‘not methylated / expressed’*** | 2906 | 6 | 745 | 1 | 3658 |
| ***‘methylated / expressed’*** | 127 | 5454 | 12 | 110 | 5703 |
| ***‘not methylated / not expressed’*** | 2705 | 1 | 12610 | 22 | 15338 |
| ***‘methylated /  not expressed’*** | 27 | 121 | 111 | 673 | 932 |
|  | 5765 | 5582 | 13478 | 806 |  |

**Table S13. Percentage of embryonic genes changing category assignment from embryo to adult.**

| embryo/adult | ***‘not methylated / expressed’*** | ***‘methylated / expressed’*** | ***‘not methylated / not expressed’*** | ***‘methylated /  not expressed’*** |  |
| --- | --- | --- | --- | --- | --- |
| ***‘not methylated / expressed’*** | 79.4% | 0.2% | 20.4% | 0.0% | 100% |
| ***‘methylated / expressed’*** | 2.2% | 95.7% | 0.2% | 1.9% | 100% |
| ***‘not methylated / not expressed’*** | 17.6% | 0.0% | 79.3% | 0.1% | 100% |
| ***‘methylated /  not expressed’*** | 2.9% | 13.0% | 11.9% | 72.2% | 100% |

**Table S14. Genes which lose at least 10% of methylation from embryos to adults and the respective GO terms.** Gene IDs from Wilhelm et al. 2025 (Colorado potato beetle gene expression atlas). GO terms: MF = Molecular function; BP = Biological process; CC = Cellular component

| **Gene ID** | **Methylation change** | **Proposed gene function (BLAST/InterPro)** | **GO terms** |
| --- | --- | --- | --- |
| LdNA_31222 | -9.9 | Prefoldin subunit 2 | GO:0006457 **BP** protein folding  GO:0016272 **CC** prefoldin complex  GO:0051082 MF unfolded protein binding |
| LdNA_4079 | -10.1 |  |  |
| LdNA_13764 | -10.5 | U1 small nuclear ribonucleoprotein 70 kDa |  |
| LdNA_3451 | -10.61 |  |  |
| LdNA_19451 | -10.66 | Large ribosomal subunit protein uL22 | GO:0003735 **MF** structural constituent of ribosome  GO:0006412 **BP** translation  GO:0015934 **CC** large ribosomal subunit  GO:0005840 **CC** ribosome |
| LdNA_1861 | -10.88 | Protein NYNRIN;  Ribonuclease H like | GO:0015074 **BP** DNA integration  GO:0003676 **MF** nucleic acid binding |
| LdNA_26425 | -10.93 |  |  |
| LdNA_6278 | -11.1 |  |  |
| LdNA_10925 | -11.25 | Gamma-secretase subunit pen-2 |  |
| LdNA_32333 | -12.35 | MADF domain profile\|MADF domain\|Alcohol dehydrogenase transcription factor Myb/SANT-like\|MADF domain |  |
| LdNA_32726 | -12.41 | CAD protein; Carbamoyl-phosphate synthetase |  |
| LdNA_19341 | -12.49 |  |  |
| LdNA_26103 | -12.78 |  |  |
| LdNA_26601 | -12.92 | DNA transposase THAP9 | GO:0004803 **MF** transposase activity  GO:0006313 **BP** DNA transposition |
| LdNA_12217 | -13.05 |  |  |
| LdNA_806 | -13.15 | Transposable element P transposase | GO:0004803 **MF** transposase activity  GO:0006313 **BP** DNA transposition |
| LdNA_15268 | -13.37 | VPS35 endosomal protein-sorting factor-like | GO:0032456 **BP** endocytic recycling |
| LdNA_26037 | -13.91 |  |  |
| LdNA_12977 | -14.00 |  |  |
| LdNA_19338 | -14.47 | RNA-binding protein 47 | GO:0003723 **MF** RNA binding  GO:0003676 **MF** nucleic acid binding |
| LdNA_21485 | -14.91 | BRICK1, SCAR/WAVE actin-nucleating complex subunit | GO:0007015 **BP** actin filament organization  GO:0031209 **CC** SCAR complex  GO:0044877 **MF** |
| LdNA_1487 | -15.02 |  |  |
| LdNA_26860 | -15.41 | Transposable element P transposase | GO:0004803 **MF** transposase activity  GO:0006313 **BP** DNA transposition |
| LdNA_14370 | -16.55 | Tigger transposable element-derived protein 2; InterPro: DDE superfamily endonuclease\|DDE superfamily endonuclease domain | GO:0003676 **MF** nucleic acid binding |
| LdNA_22231 | -18.18 |  |  |
| LdNA_26356 | -18.546 | Hermes dimerisation domain\|Hermes transposase DNA-binding domain\|Hermes transposase, DNA-binding domain |  |
| LdNA_34123 | -18.6 | Extracellular Endonuclease, subunit A\|DNA/RNA non-specific endonuclease superfamily | GO:0003676 **MF** nucleic acid binding  GO:0016787 **MF** hydrolase activity  GO:0046872 **MF** metal ion binding |
| LdNA_20200 | - 20.36 | Selenoprotein F |  |
| LdNA_19457 | - 25.56 | Large ribosomal subunit protein uL22 | GO:0003735 **MF** structural constituent of ribosome  GO:0006412 **BP** translation  GO:0015934 **CC** large ribosomal subunit  GO:0005840 **CC** ribosome |
| LdNA_26712 | - 26.94 |  |  |

## Supplementary Figures


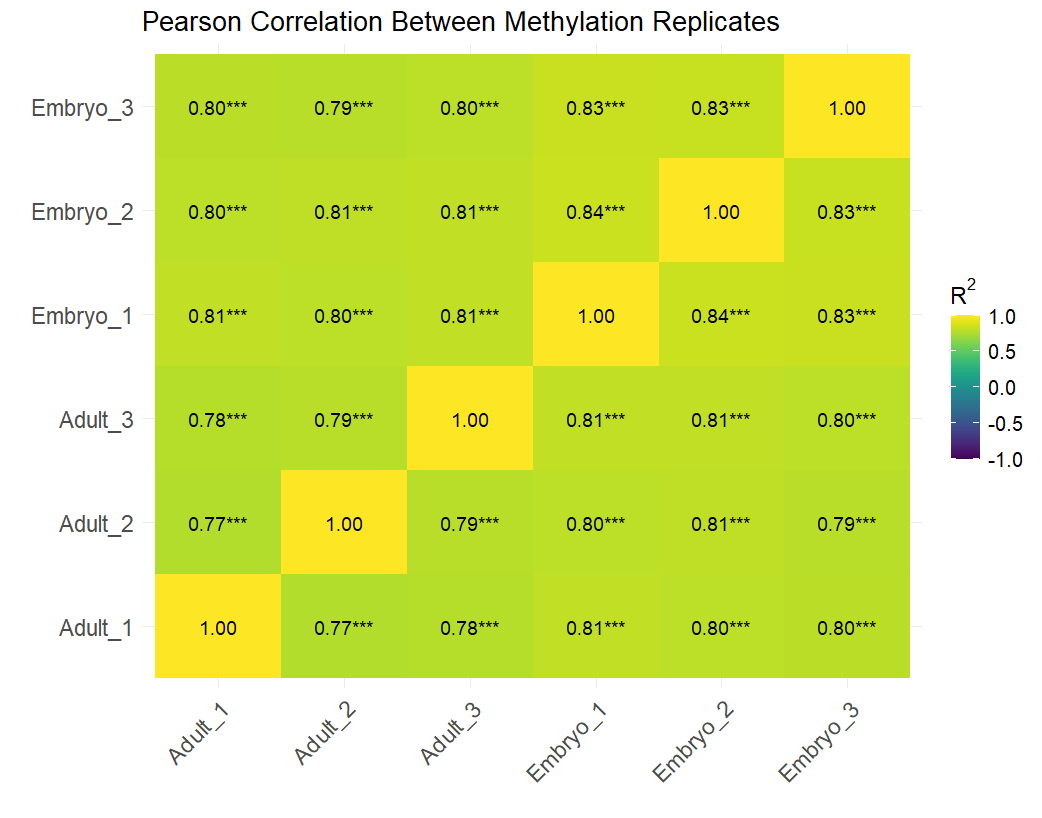


**Figure S1. Correlation matrix showing the Pearson correlation coefficients between biological replicates and life stages of *L. decemlineata* based on genomic CpG sites.** Pearson correlation coefficients (R^2^) were calculated for the methylation levels per CpG site. Significant correlations (p < 0.05) are marked with stars. The correlation strength is color coded according to the legend, with yellow and green shades indicating stronger positive correlations.

**
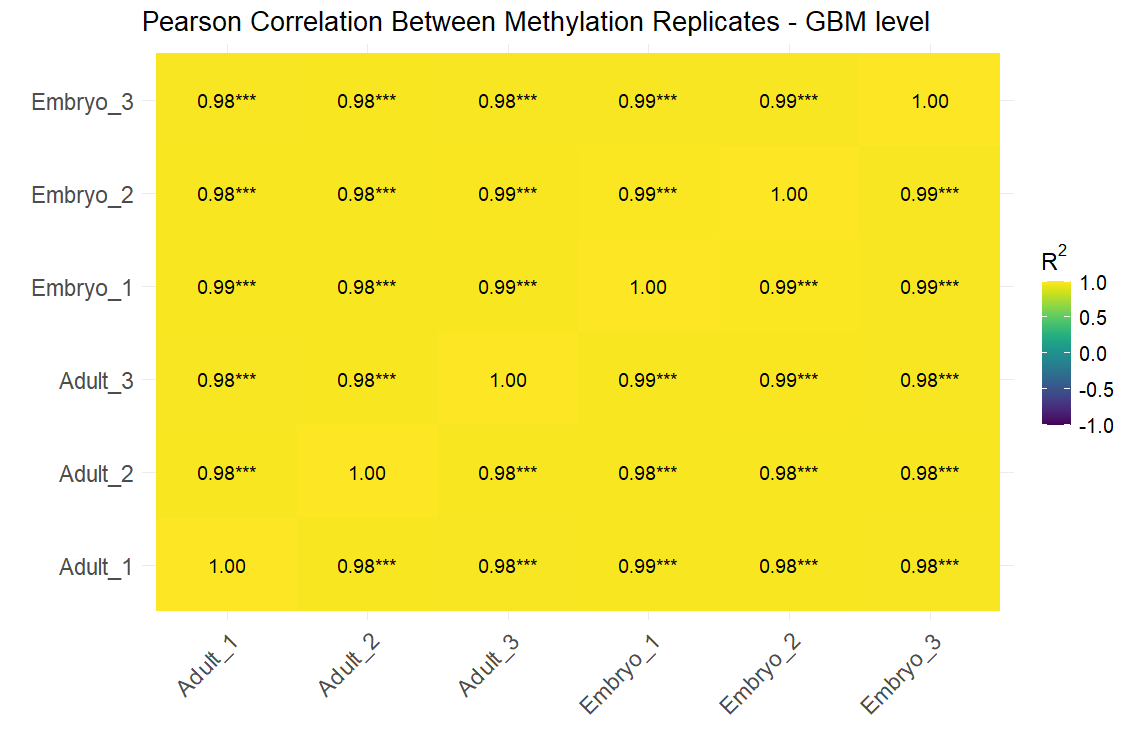
**

**Figure S2. Correlation matrix showing the Pearson correlation coefficients between biological replicates and life stages of *L. decemlineata* based on genes.** Pearson correlation coefficients (R^2^) were calculated for the mean % CpG methylation per gene. Significant correlations (p < 0.05) are marked with stars. The correlation strength is color-coded according to the legend, with yellow and green shades indicating stronger positive correlations.


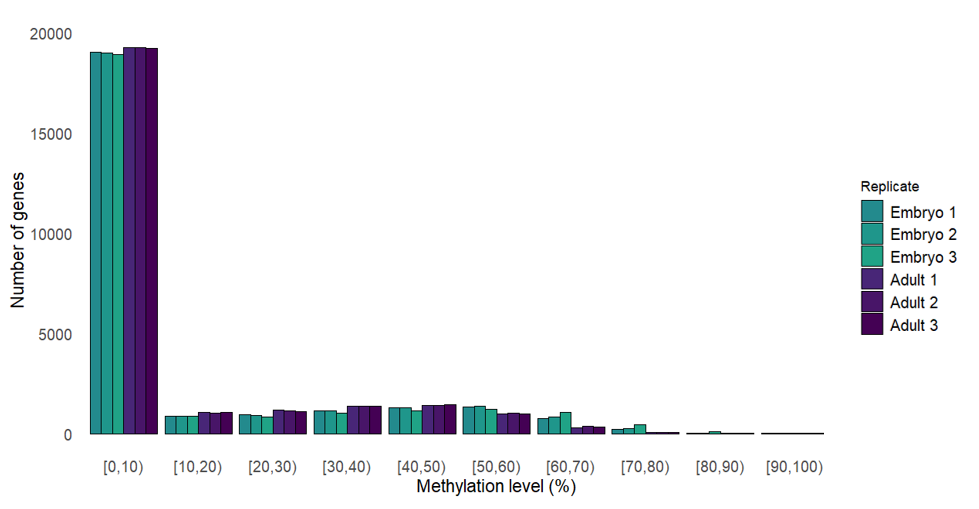


**Figure S3. Number of genes in each methylation percentage range for each replicate of embryo and adult *L. decemlineata***.


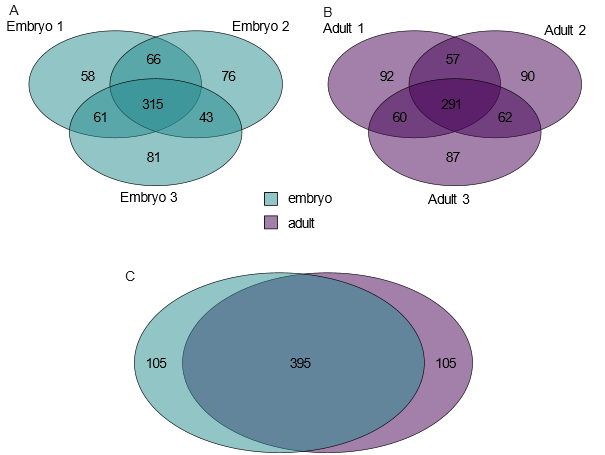


**Figure S4. Overlap of the 500 genes with the highest gene body methylation (%) in *L. decemlineata* between**

**A) the embryonic replicates.** One embryonic replicate consists of 30 pooled embryos of the same age (+- 1h).

**B) Between adult replicates and C) between embryos and adults.** The mean methylation level of the 3 replicates of each life stage was used.


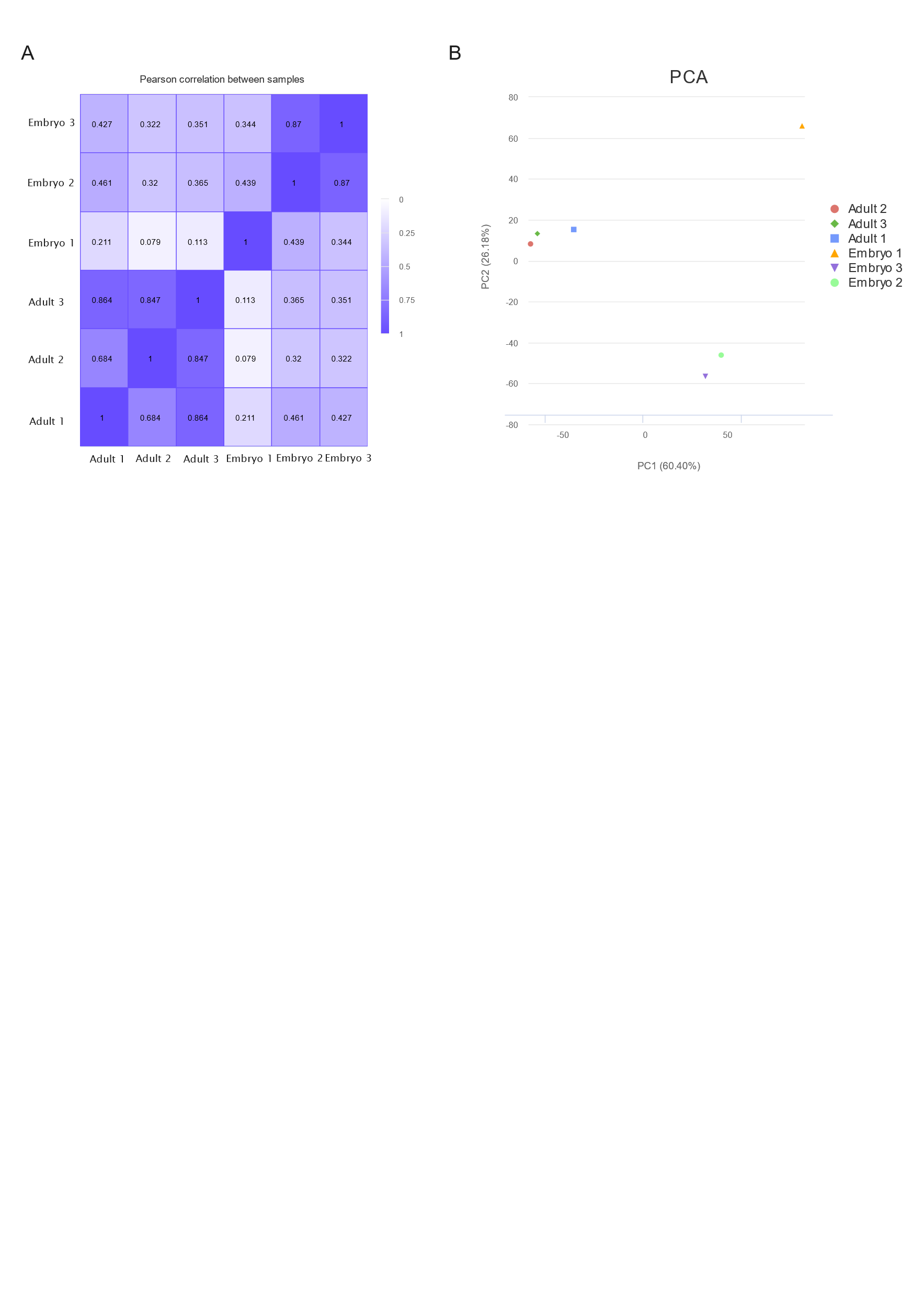


**Figure S5. A) Pearson correlation and B) Principal Component Analysis (PCA) of the 3 embryonic and 3 adult *L. decemlineata* RNA-seq replicates.** Embryo 1 does not correlate or cluster well with Embryo 2 and Embryo 3.


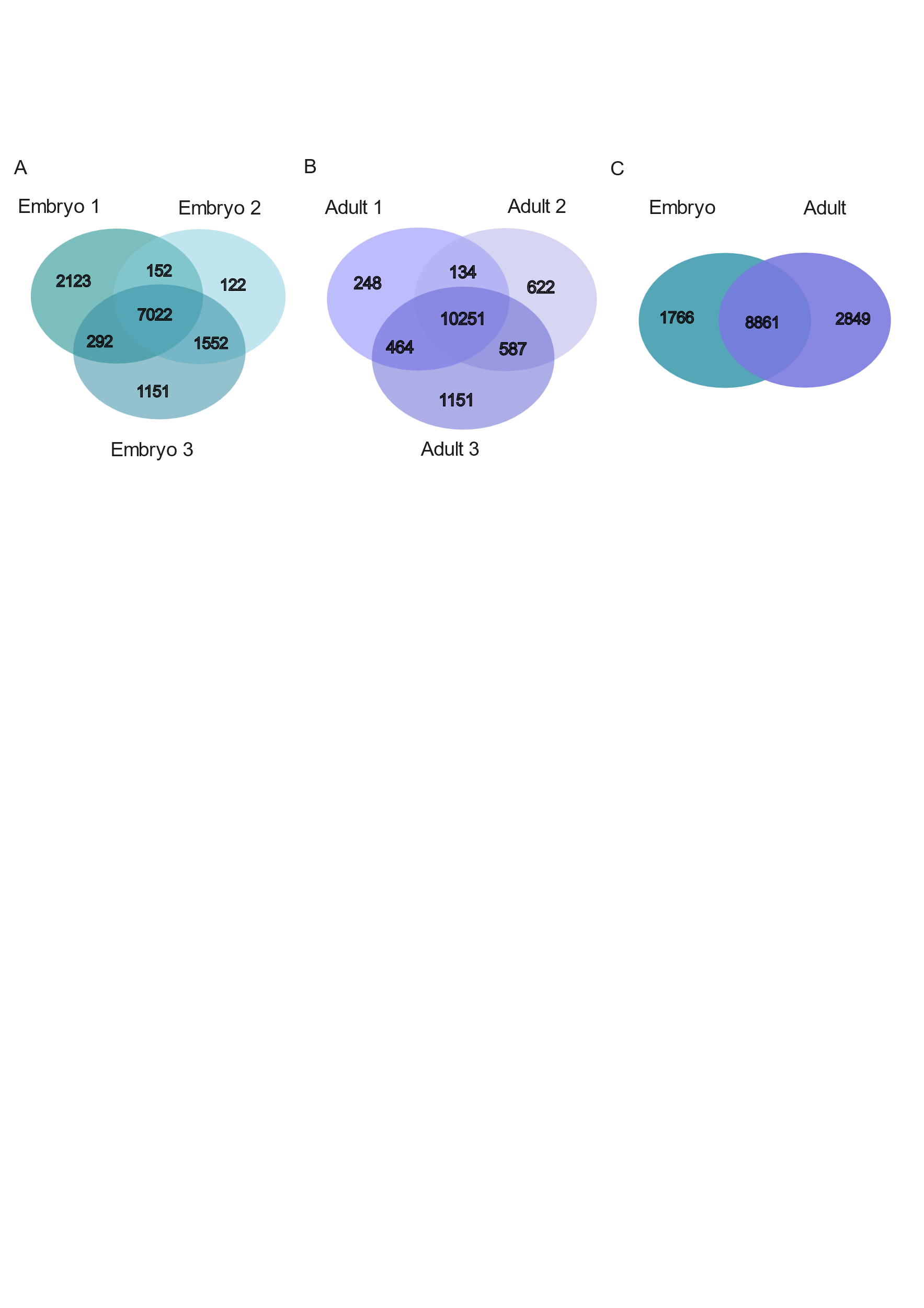


**Figure S6. Venn diagrams for *L. decemlineata* RNA-seq replicates. A) Embryo.** Shown are the numbers of expressed genes shared between the replicates. Replicate Embryo 1 shows little overlap with the other embryonic replicates. As it also did not cluster well with the other two embryonic replicates, it was removed from further analyses. **B) Adult.** Shown are the numbers of expressed genes shared between the replicates **C) Comparison of embryo and adult.** Shown are the numbers of expressed genes shared between the life stages


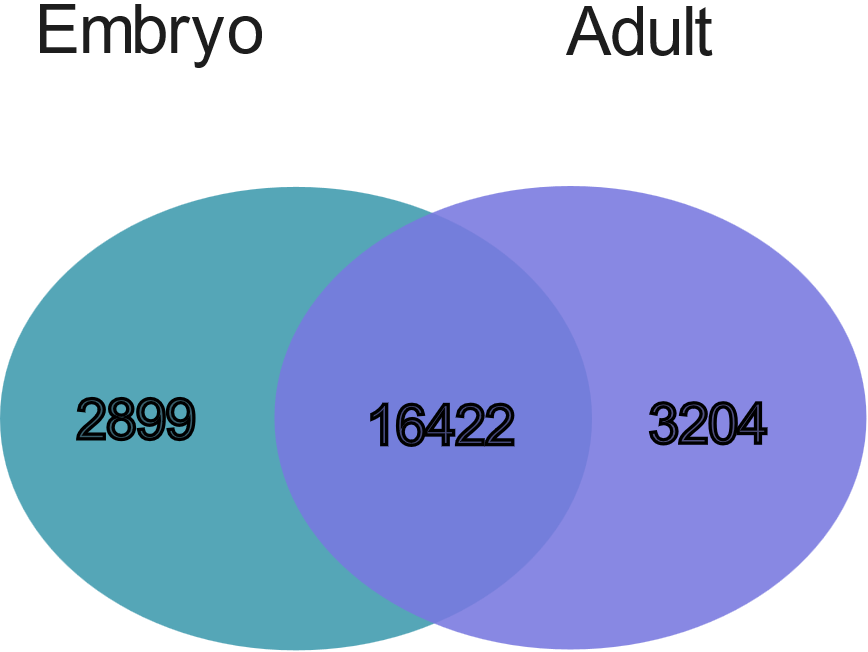


**Figure S7. Comparison of Adult and Embryo *L. decemlineata* RNA-seq replicates after removal of Embryo 1.** Shown are the numbers of expressed genes shared between the life stages


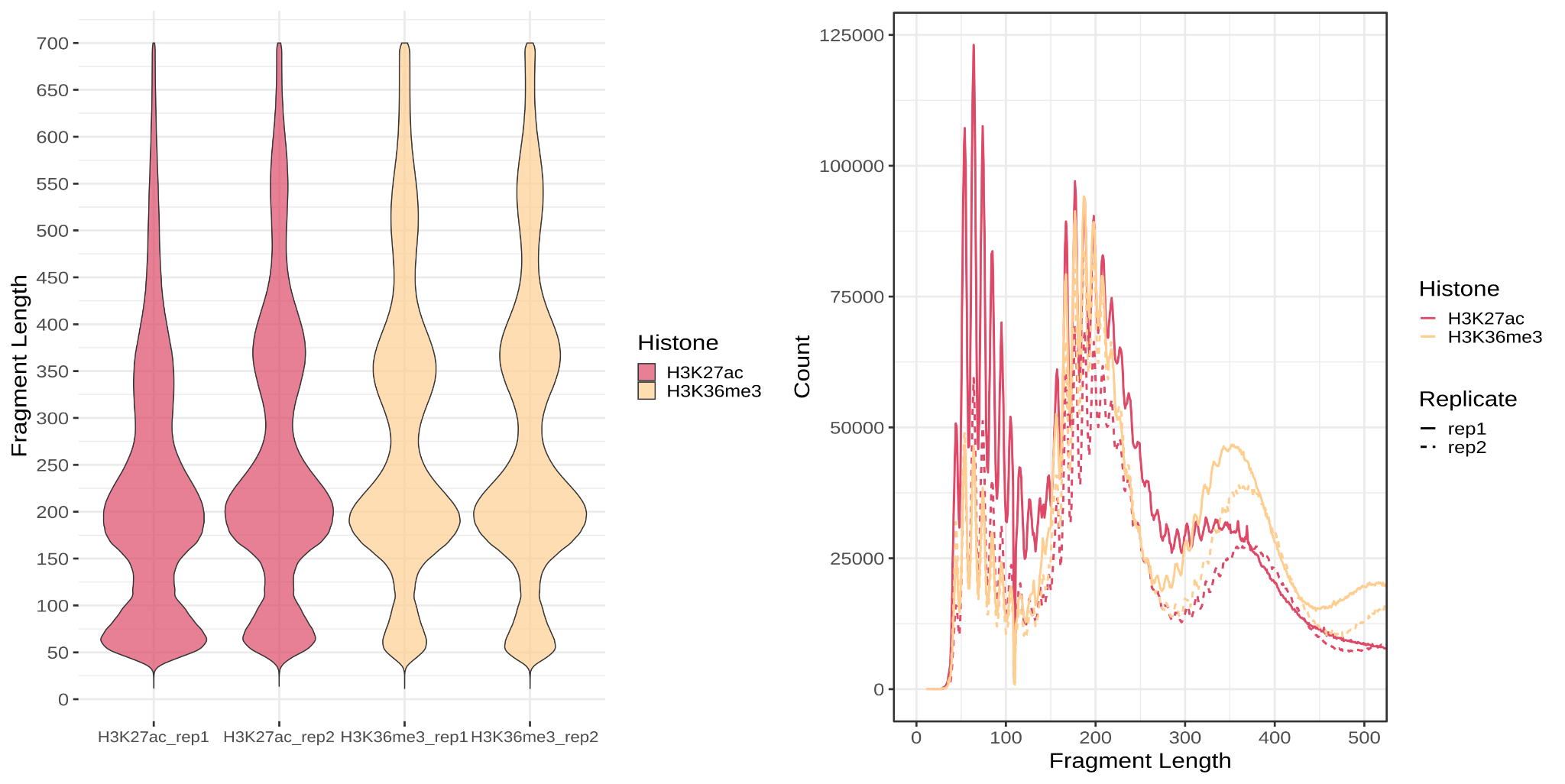


**Figure S8. Fragment length distribution and fragment length on single-base pair resolution for validating CUT&Tag results.** Duplicate reads were not removed.


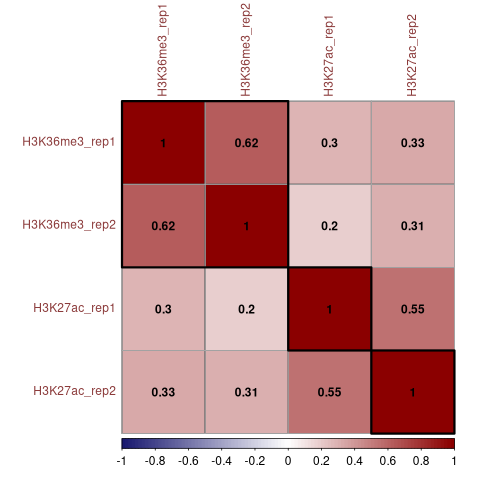


**Figure S9. Correlation matrix displaying Pearson correlation coefficients between biological replicates of embryonic histone marks.** To evaluate reproducibility, the genome was divided into 500 bp bins, and log2-transformed read counts were computed for each bin across replicates. The strength of correlation is color-coded, with darker red shades representing stronger positive correlations.


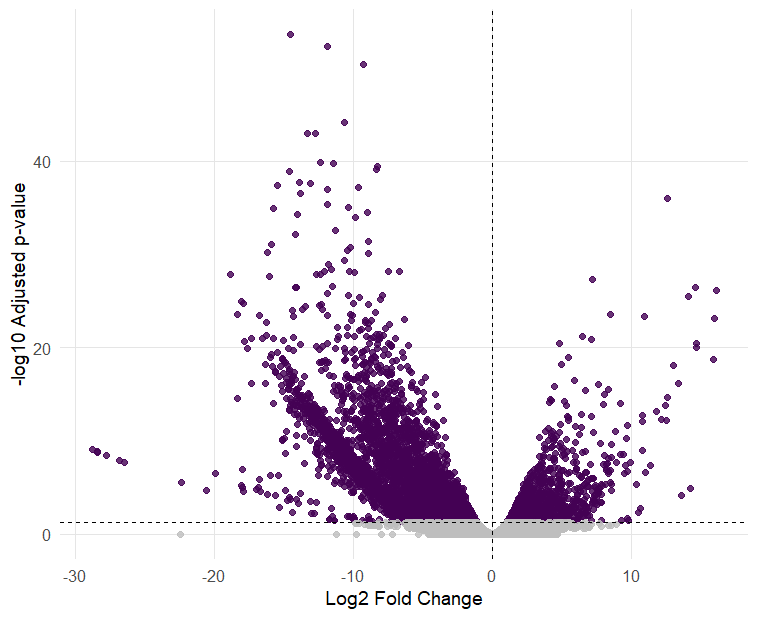


**Figure S10. Volcano plot for differentially expressed genes. Adjusted p-value vs. the log2FC of gene expression was plotted.** Dashed horizontal line indicates the significance threshold for the log2FC (1). Color indicates significant adjusted p-value. Higher expression in adults is indicated by data points on the right side.


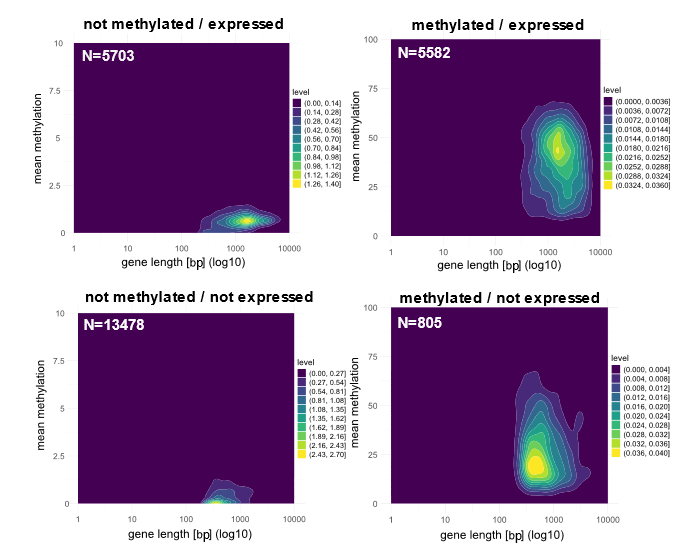


**Figure S11. Relationship between gene length and mean methylation (%) in different categories in adult *L. decemlineata.*** (Note difference in scale). The color gradient (level) represents the density of genes at different methylation levels, with brighter shades indicating regions of higher density.

**
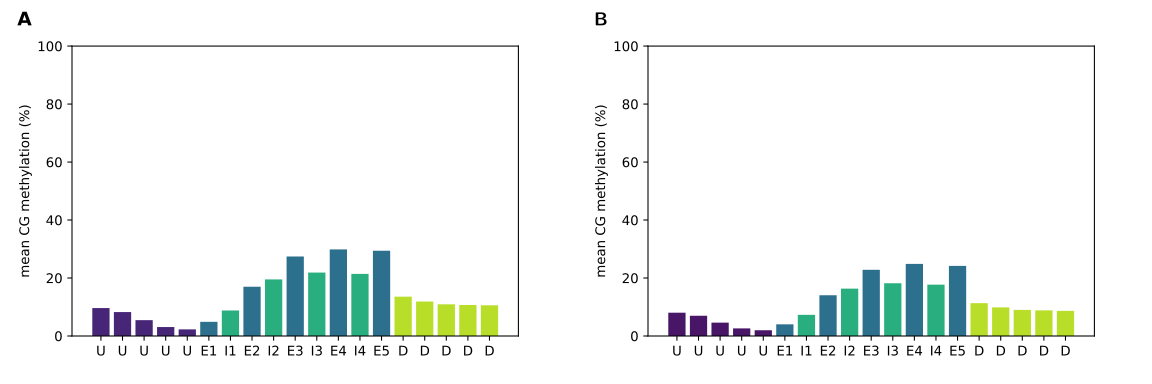
**

**Figure S12. Methylation level of different genic segments, in A) embryo and B) adult.** Shown is the mean methylation level of different genic segments across all annotated *L. decemlineata* genes. E1 to E5 represent the first 5 exons; I1-I4 represent the first four introns; U – upstream region; D – downstream region.


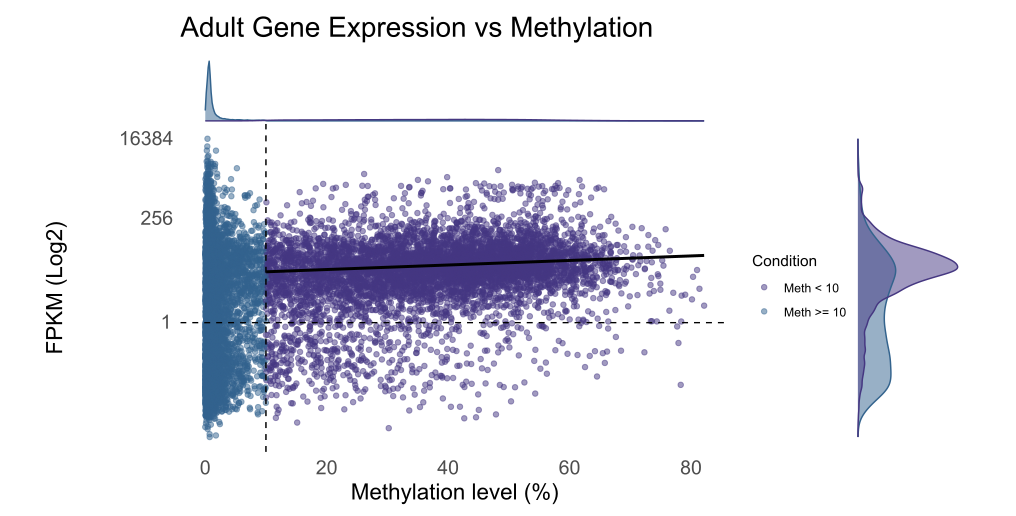


**Figure S13. Association between gene expression level (Log FPKM) and gene body methylation level in adults.** Dashed gray lines indicate thresholds used in methylation and FPKM. All values that equal 0 were removed.


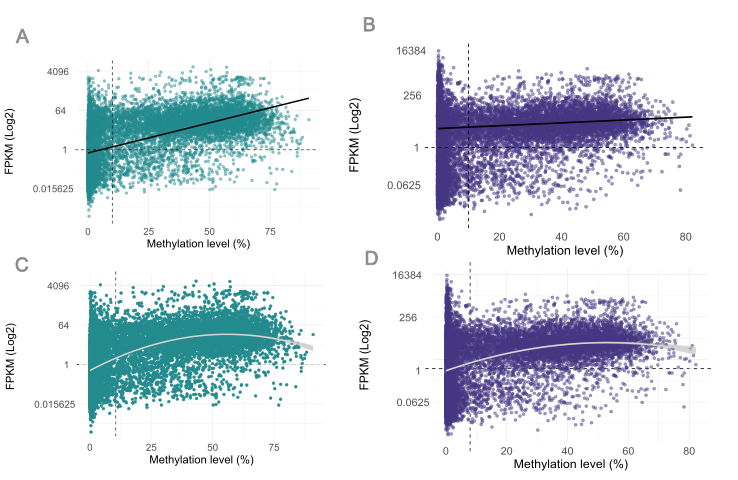


**Figure S14. Association between gene expression level (Log FPKM) and gene body methylation level in *L. decemlineata* linear and square regression.** Dashed gray lines indicate thresholds used in methylation and FPKM. Square regression was calculated for all values in A) embryo R2: 0.02534; df: 14873; p-value: 0.000591 and B) adult R2: 0.00066; df: 16197; p-value 0.001753 in C) embryo R2: 0.002757; df: 5700; p-value: 0.117161 n.s. and D) adult R2: 0.00444; df: 5579; p-value: 0.779 n.s. For *‘methylated / expressed’* genes, while the linear component of methylation is significantly positively associated with gene expression (p < 0.001), the quadratic term is not (embryo p-value: 0.117161; adult p = 0.779), indicating no curved relationship. The model explains only a small portion of the variance in gene expression (R² = 0.48%).


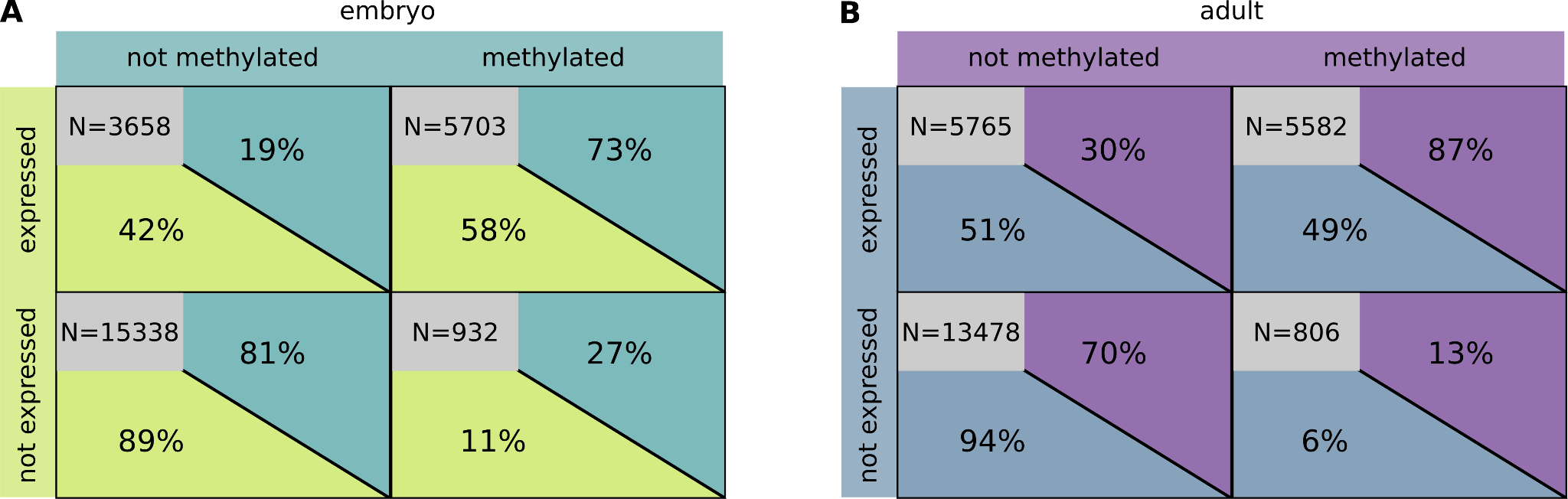


**Figure S15. Matrix summarizing the relationship between methylation and expression status in embryos and adults.** (Example upper left corner (embryo): 42% of expressed genes are not methylated, while only 19% of not methylated genes are expressed.). Numbers of genes that are changing categories between embryos and adults can be found in Table S12 and S13.


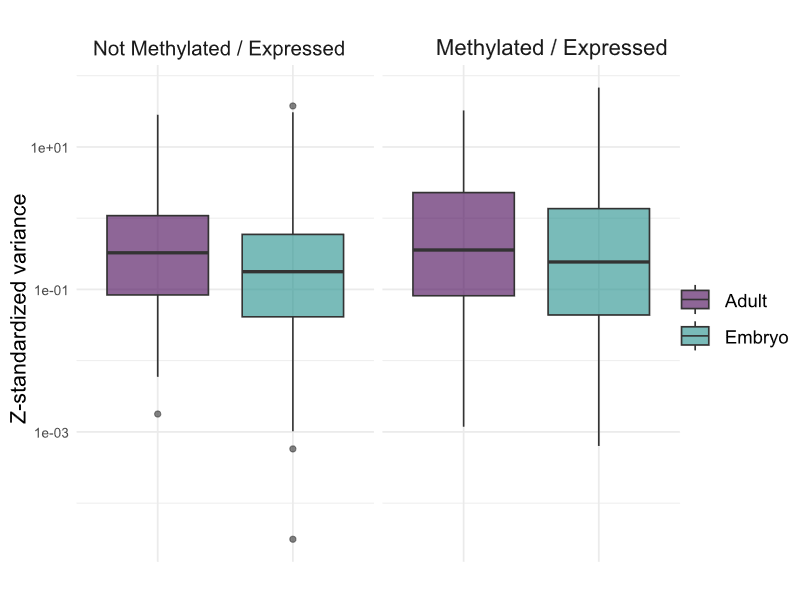


**Figure S16. Z-normalized expression variance between replicates of the embryonic subsets of ‘*not methylated / expressed*’ and ‘*methylated / expressed*’.** Levene's test for homogeneity of variance revealed no significant difference between the subsets.


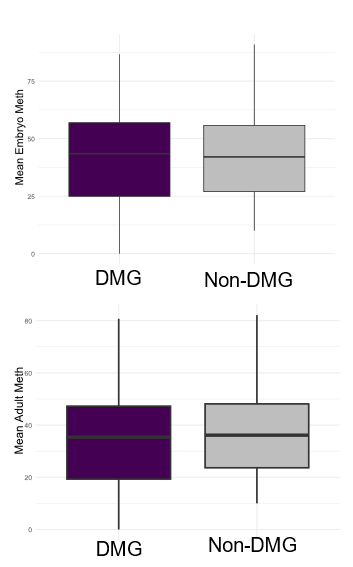


**Figure S17. Methylation levels of genes with differentially methylated regions (DMRs) between embryos and adults, compared to genes without differential methylation across stages.**


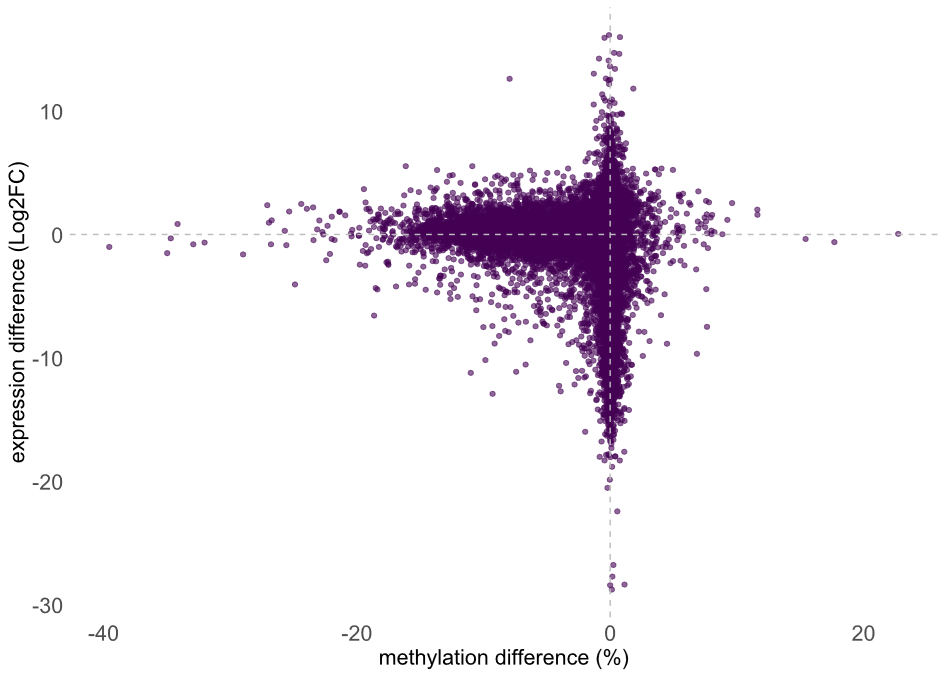


**Figure S18. Relationship of gene body methylation and gene expression between embryo and adult.** Shown is the correlation of differences in gene body methylation and gene expression (log2 fold change [Log2FC]) between embryonic and adult life stages. R^2^: 0.051, df: 17546, adj. P-value < 2.2e -16


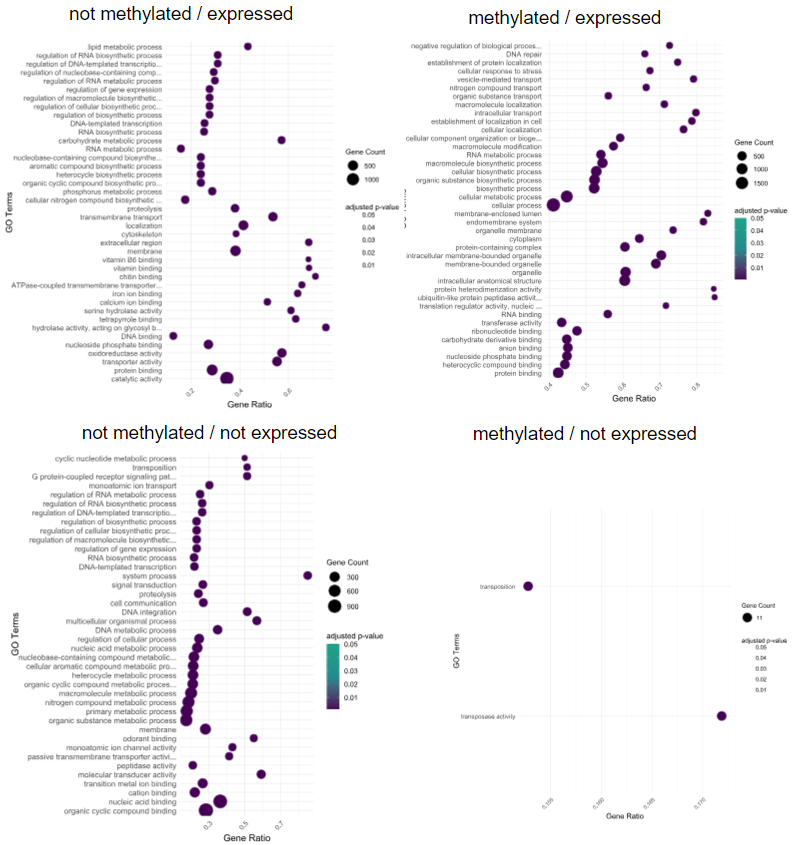


**Figure S19. Adult top 40 significantly enriched Gene Ontology (GO) terms for the four subsets.** The p-value was adjusted using the Benjamini-Hochberg procedure. Gene count is the number of genes associated with a GO term, whereas ‘Gene Ratio’ is the percentage of genes of the specific subset in the given GO terms. Categories vary in their number of genes included, resulting in different numbers of enriched GO terms. (‘*not methylated / expressed*’ = 135 GO; ‘*methylated / expressed*’ = 123 GO; ‘*not methylated / not expressed*’ = 120 GO; ‘*methylated / not expressed*’ = 2 GO).

**
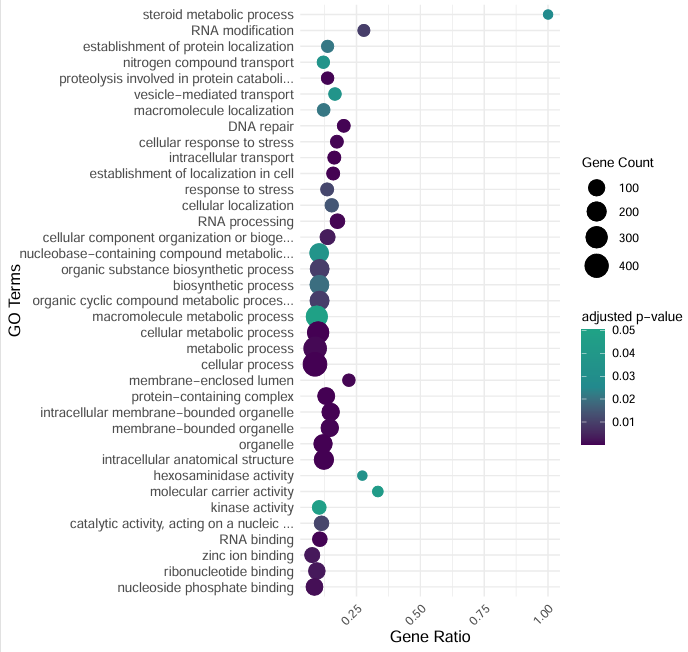
**

**Figure S20. Top 40 significantly enriched Gene Ontology (GO) terms for those genes changing more than 10% of methylation between embryos and adults.** The p-value was adjusted using the Benjamini-Hochberg procedure. Gene count is the number of genes associated with a GO term, whereas ‘Gene Ratio’ is the percentage of genes of the specific subset in the given GO terms. This gene set consists of 1359 hypomethylated and 5 hypermethylated genes.


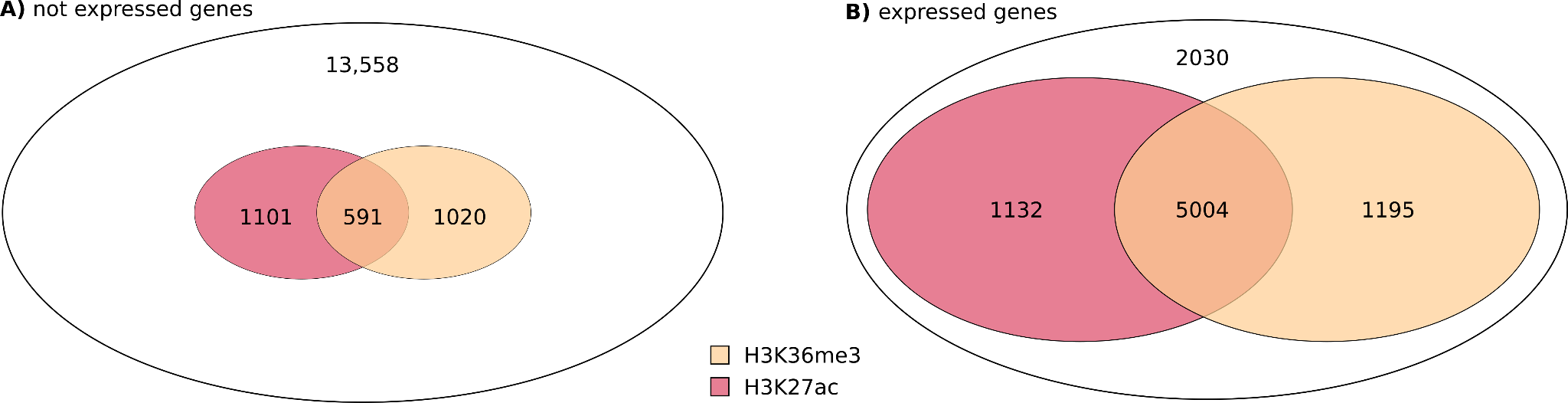


**Figure S21. Venn diagrams showing the proportion of genes with enrichment of H3K27ac, H3K36me3 or both in A) not expressed genes.** Total number of not expressed genes = 16,270; percentage of genes carrying at least one histone mark: 16.66%; percentage of genes with H3K27ac enrichment: 10.39%; percentage of genes with H3K36me3 enrichment: 9.9%; percentage of genes with enrichment of both marks: 3.63%. **B) expressed genes.** Total number of expressed genes = 9,361; percentage of genes carrying at least one histone mark: 78.31%; percentage of genes with H3K27ac enrichment: 65.53%; percentage of genes with H3K36me3 enrichment: 66.22%; percentage of genes with enrichment of both marks: 53.45%.

**
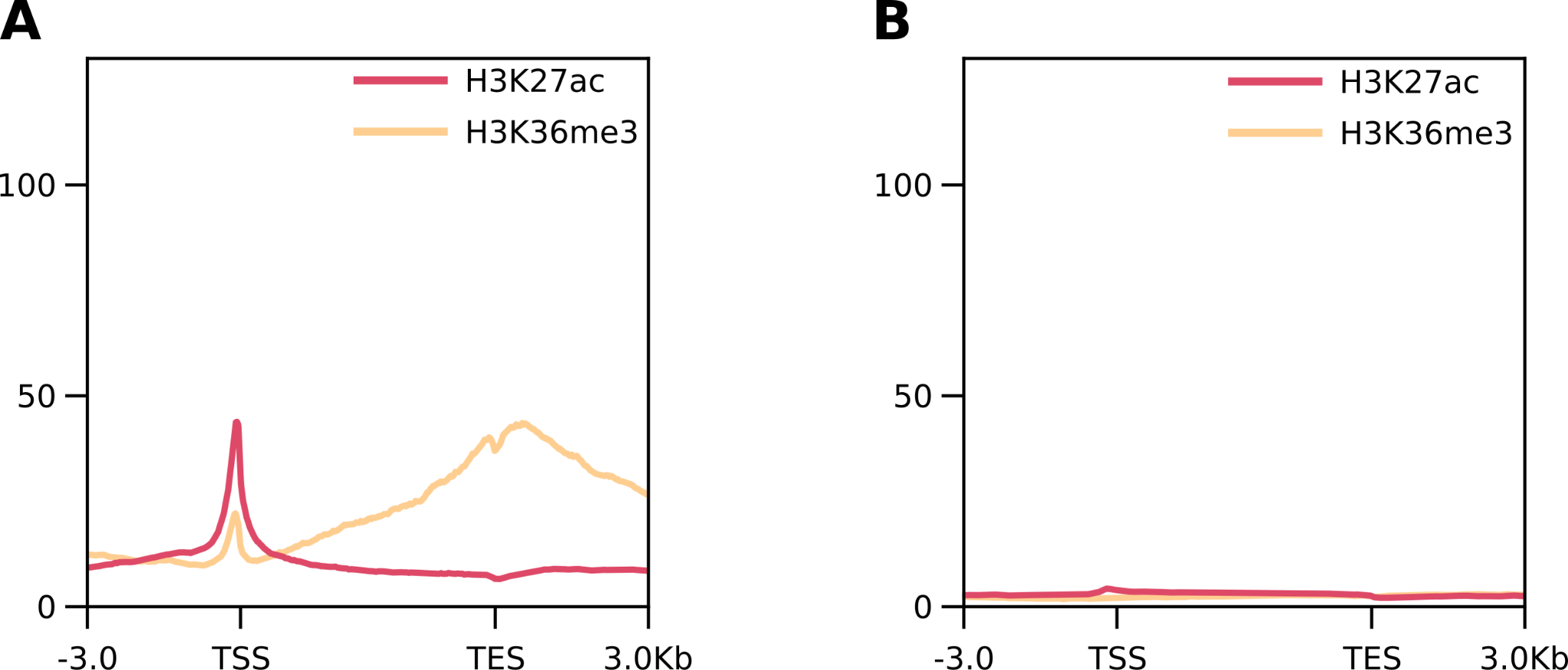
**

**Figure S22. Enrichment patterns of H3K27ac and H3K36me3 in A) ‘expressed’ and B) ‘not expressed’ genes.** Shown are the profiles for replicate 2.

**
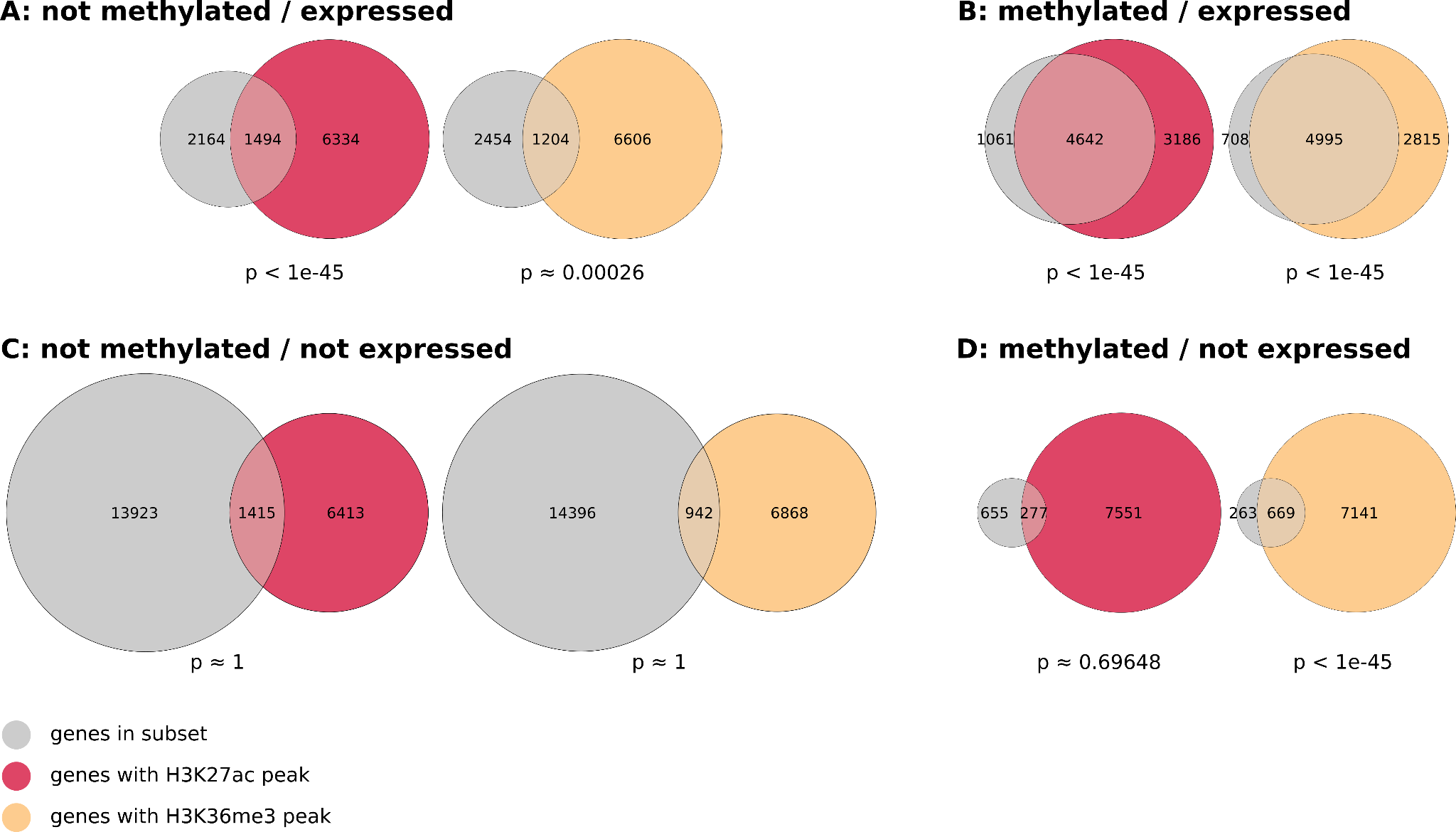
**

**Figure S23. Area-adjusted Venn diagram showing the overlap between the genes in the four subsets and genes marked with histone modifications.** Total number of genes with H3K27ac peak: 7828. Total number of genes with H3K36me3 peak: 7810. Total number in each subset: A) 3658, B) 5703, C) 15338, D) 932. Below each Venn diagram, the p-value for the respective overlap is shown.


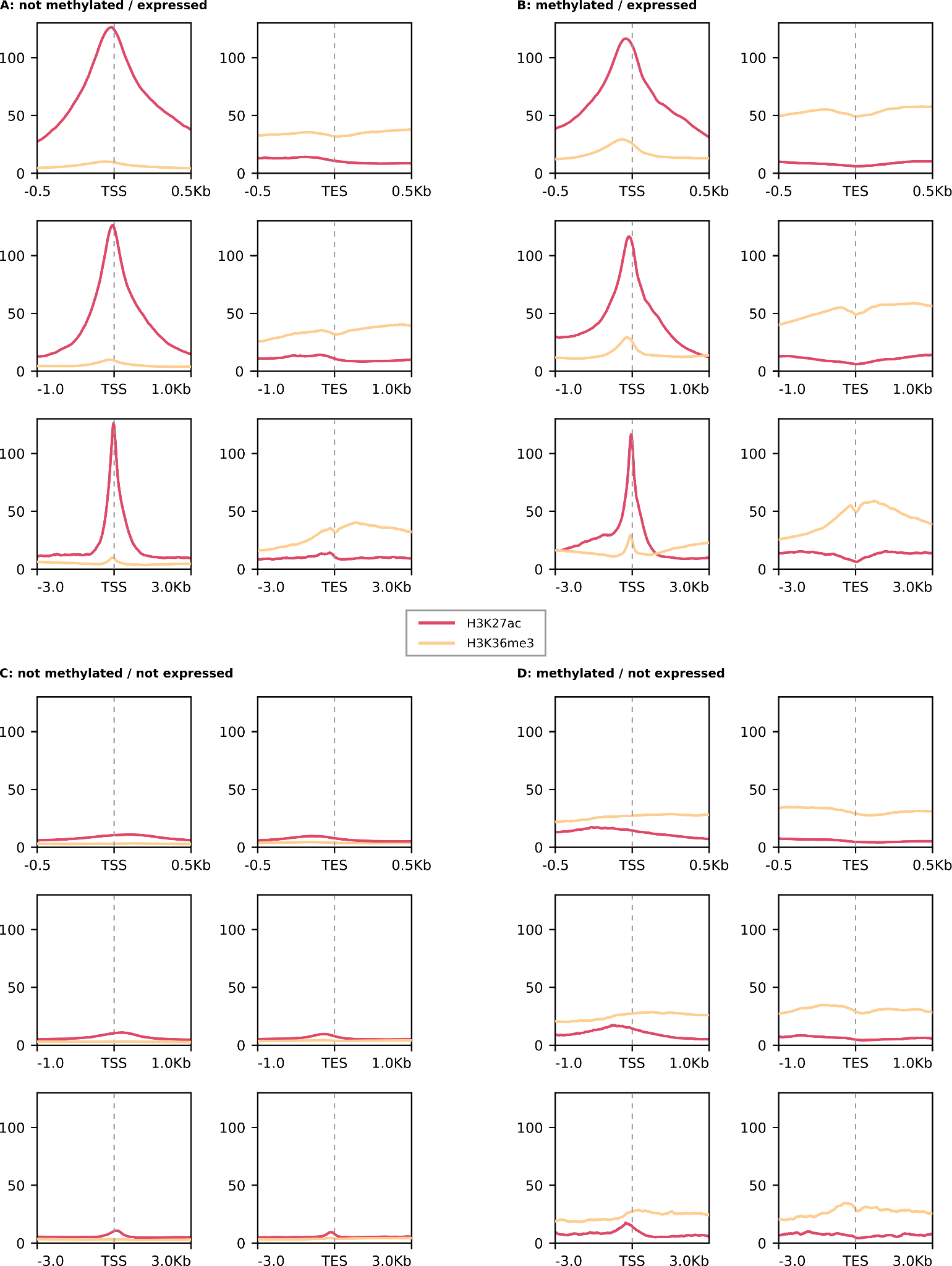


**Figure S24. Enrichment profiles of the H3K27ac and H3K36me3 distribution transcription start site**

**(TSS) and transcription end site (TES).** Flanking regions of 0.5 kb, 1 kb and 3 kb were used. Shown are the

profiles of replicate 1.

**
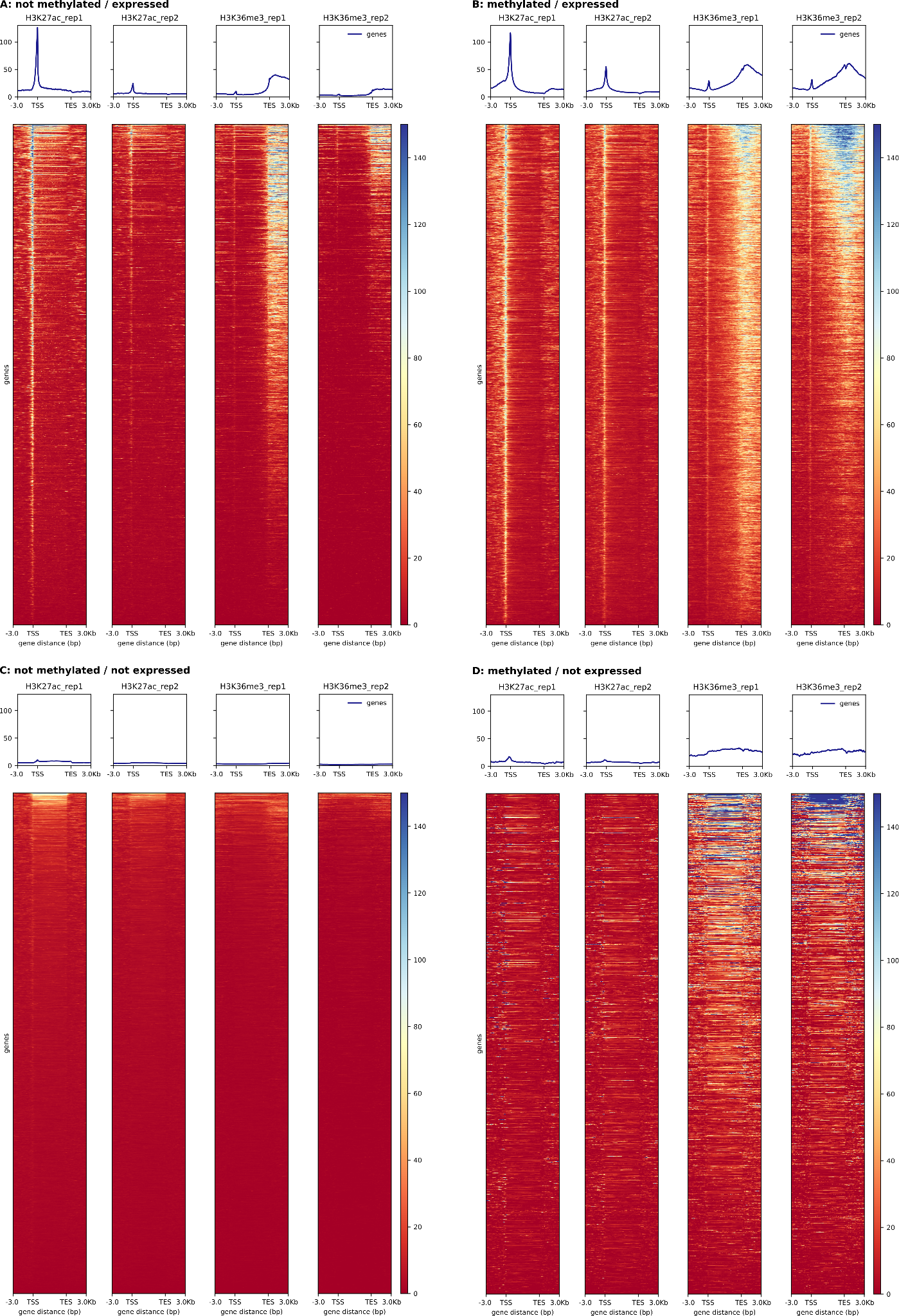
**

**Figure S25. Profiles and corresponding heatmaps of H3K27ac and H3K36me3 enrichment in gene bodies for each replicate.** Genes are normalized to a length of 5 kb, with 3 kb upstream and downstream of TSS and TES, respectively.
